# Using digital surveillance tools for near real-time mapping of the risk of infectious disease spread

**DOI:** 10.1038/s41746-021-00442-3

**Published:** 2021-04-16

**Authors:** Sangeeta Bhatia, Britta Lassmann, Emily Cohn, Angel N. Desai, Malwina Carrion, Moritz U. G. Kraemer, Mark Herringer, John Brownstein, Larry Madoff, Anne Cori, Pierre Nouvellet

**Affiliations:** 1 MRC Centre for Global Infectious Disease Analysis, School of Public Health, Imperial College London, Faculty of Medicine, London, UK; 2 ProMED, International Society for Infectious Diseases, Brookline, MA USA; 3 Computational Epidemiology Group, Division of Emergency Medicine, Boston Children’s Hospital, Boston, MA USA; 4 Department of Health Science, Sargent College, Boston University, Boston, MA USA; 5 Department of Zoology, Tinbergen Building, Oxford University, Oxford, UK; 6 Department of Pediatrics, Harvard Medical School, Boston, USA; 7 healthsites.io, London, UK; 8 School of Life Sciences, University of Sussex, Brighton, UK

**Keywords:** Infectious diseases, Statistical methods

## Abstract

Data from digital disease surveillance tools such as ProMED and HealthMap can complement the field surveillance during ongoing outbreaks. Our aim was to investigate the use of data collected through ProMED and HealthMap in real-time outbreak analysis. We developed a flexible statistical model to quantify spatial heterogeneity in the risk of spread of an outbreak and to forecast short term incidence trends. The model was applied retrospectively to data collected by ProMED and HealthMap during the 2013–2016 West African Ebola epidemic and for comparison, to WHO data. Using ProMED and HealthMap data, the model was able to robustly quantify the risk of disease spread 1–4 weeks in advance and for countries at risk of case importations, quantify where this risk comes from. Our study highlights that ProMED and HealthMap data could be used in real-time to quantify the spatial heterogeneity in risk of spread of an outbreak.

## Introduction

Recent epidemics and the ongoing COVID-19 pandemic underscore the importance of understanding the risk of infectious disease spread in real-time to support public health prevention and containment measures. The emergence of novel infectious diseases has accelerated over the past several decades as a result of significant changes in land use, population growth, and increasing international travel and trade^1,2^. Outbreaks that begin in the most remote parts of the world can now spread swiftly to urban centres and to countries far away with global consequences^3^. A potent example of this phenomenon is the ongoing COVID-19 pandemic that originated in China, and has spread to practically every country and region around the world at the time of writing^4^.

Outbreak analysis and real-time modelling have provided key inputs to inform public health response in past outbreaks and pandemics^5–7^ as well as during the ongoing COVID-19 pandemic^8–10^. Critical to model performance is the quality of data incorporated into forecasting algorithms. Reliable data streams that are available in real-time to inform risk assessments are therefore crucial^11^. While data on the number of cases and deaths collected through traditional surveillance, for example via existing public health infrastructure, are robust, collecting these data is resource intensive, and the data are therefore only available for upstream analysis after considerable delay^12^.

Internet-based disease detection and monitoring tools offer real-time and rapid data dissemination on emerging infectious diseases around the world. Digital disease surveillance is less costly and time consuming compared to traditional surveillance. However the data acquired are subject to more noise than those collected by traditional public health surveillance. Digital surveillance tools are now recognised as important adjunct tools to traditional disease surveillance in the rapid recognition and monitoring of emerging infectious disease threats^13,14^. While there has been a growing interest in using various internet-based data streams for epidemiological investigations^15–18^, to date, no automated framework that combines data streams, analyses them in a statistically robust manner, and produces actionable reports in near real-time has been developed.

The Program for Monitoring Emerging Diseases (ProMED, www.promedmail.org) was one of the first entrants in the field of digital disease surveillance^19^. ProMED collates information from formal and informal sources including media reports, official reports, social media, local observers, and a network of clinicians throughout the world. Reports generated through bottom-up surveillance are reviewed, vetted, and commented on by a team of subject matter experts before being posted to the ProMED network. ProMED reports reach more than 80,000 followers in at least 185 countries multiple times per day^20^. Outbreak analysts and subject matter experts at ProMED have raised timely alarms about major outbreaks in the past, such as warning about the spread of Zika to the Americas^21^, or early detection of SARS^22^. ProMED has been internationally recognised for providing the first detailed report and risk assessment of a cluster of pneumonia cases of unknown aetiology in Wuhan, China in December 2019 leading to the COVID-19 pandemic^23^.

HealthMap (www.healthmap.org) is another widely used tool for disease outbreak monitoring. In addition to ProMED alerts, HealthMap utilises online news aggregators, eyewitness reports and other formal and informal sources of information and allows for visualisation of alerts on a map^24^. The surveillance data collected by HealthMap and ProMED has been incorporated into the Epidemic Intelligence from Open Sources (EIOS) surveillance system, developed by the World Health Organization (WHO). Both ProMED and HealthMap are used by key public health bodies, including the US Centres for Disease Control and Prevention (CDC) and the WHO.

In this study, we propose a new statistical framework to estimate and visualise risks posed by outbreak events. Our approach integrates multiple data streams to quantify spatial heterogeneity in the risk of disease spread. A secondary objective is to explore the potential of such framework to forecast the future incidence trajectory. We report a retrospective analysis of the 2013–16 West African Ebola epidemic using the data curated by ProMED and HealthMap. To assess the robustness of this digital surveillance data, we also applied the framework retrospectively to data collated by the WHO that was made available at the end of this epidemic. We present a comparison of the near real-time ProMED and HealthMap data with the retrospective WHO data and the results of the spatio-temporal analysis carried out on these three data sources. A key output of our model is the quantification of the risk of the spread of an outbreak, and for each country where that risk comes from. Our analysis based solely on epidemic data available through ProMED/HealthMap provides a realistic appraisal of their strengths and weaknesses.

### Results

#### Comparison of ProMED, HealthMap and WHO data

**Fig. 1:**
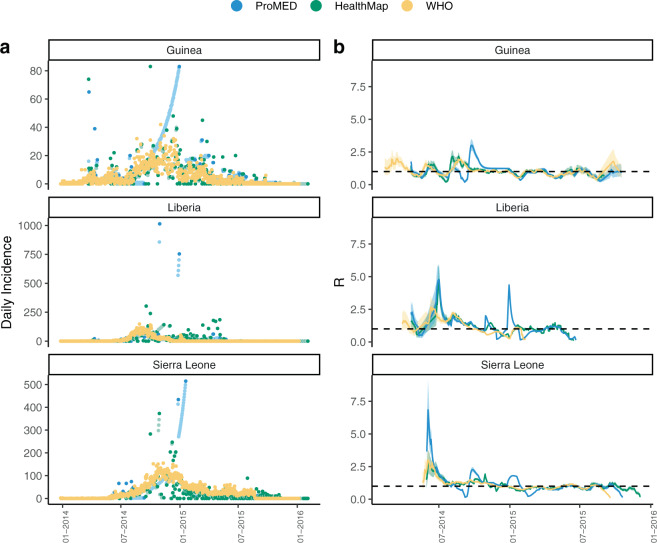
Comparison of incidence trends and R estimates Comparison of national daily incidence trends and R estimates from ProMED, HealthMap and WHO data for Guinea, Liberia and Sierra Leone. **a** Daily incidence derived from ProMED (blue), HealthMap (green) and WHO data (orange). Daily incidence that were not directly available from ProMED and HealthMap data and which were therefore imputed (see Methods) are shown in lighter shade of blue and green, respectively. WHO data were aggregated to country level. The y-axis differs for each plot. **b** The median time-varying reproduction number *R*^*t*^ estimated using the WHO data (orange), ProMED (blue) and HealthMap (green) data. The shaded regions depicts the 95% credible intervals (95% CrI) for the *R*^*t*^ estimates. The reproduction number was estimated on sliding windows of 28 days with a Gamma prior with mean 1 and variance 0.25, using the R package EpiEstim^26^. Estimates shown at time *t* are for the 28-day window finishing on day *t*.

Incidence time series were computed from ProMED, HealthMap and WHO data for the three mainly affected countries, Guinea, Liberia and Sierra Leone, and are shown in Fig. 1 (see Supplementary Fig. 1 for an example of the pre-processing workflow).

We measured the correlation between the daily and weekly incidence time series derived from ProMED, HealthMap and WHO data using Pearson’s correlation coefficient. There were substantial differences between the daily incidence time series derived from the three data sources, particularly at the peak of the epidemic. However, despite these discrepancies, the weekly incidence derived from ProMED and HealthMap was moderately to highly correlated with that reported by WHO later (Pearson’s correlation coefficients aggregated across the three countries 0.44 and 0.74, respectively, *p* value < 0.001, also see Supplementary Fig. 2 for weekly trends).

To broadly assess the extent to which any differences in incidence would impact the quantification of transmissibility throughout the epidemic, we estimated the time-varying transmissibility, measured by the reproduction number *R*^*t*^ (the average number of secondary cases at time *t* per infected individual^25^), using the incidence from each of the three data sources (Fig. 1). *R*^*t*^ was estimated independently for each country using the R package EpiEstim^26^ over a sliding time window of 4 weeks. There were substantial differences in the estimates of *R*^*t*^ according to the incidence data source used (Fig. 1b). The correlation between the median *R*^*t*^ estimates from ProMED or HealthMap data with the estimates from WHO data varied from weak (0.30, between reproduction number from WHO and ProMED in Guinea) to very strong (0.72, between reproduction number from WHO and ProMED in Sierra Leone, Supplementary Fig. 3).

#### Risk of spatial spread

A spatially explicit branching process model (see Methods) was used to forecast short-term future incidence and predict the presence or absence of cases in a country. For each week and each country in Africa, our model generated an alert if the predicted incidence (using a predetermined percentile of the forecast interval) was greater than 0. We classified an alert for a given week as a true alert when the observed incidence was also greater than 0, as a false alert when no cases were observed, and as a missed alert if cases were observed but were not predicted by the model. Using different percentiles of the forecast (e.g., the median or the 95th percentile) yielded different rates of true, false and missed alerts, which were summarised in a ROC curve.

**Fig. 2:**
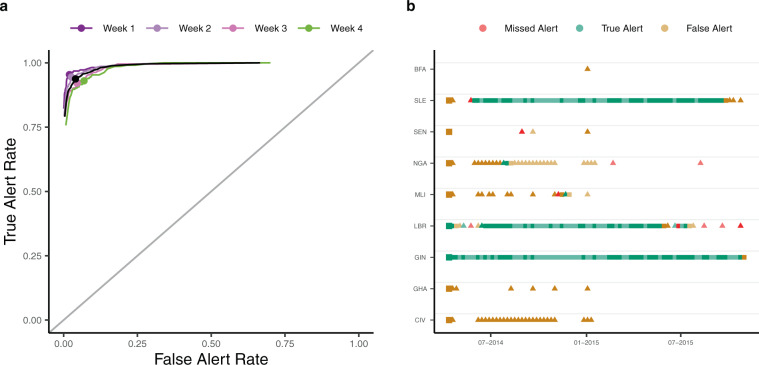
Predicted weekly presence of cases in each country. **a** The True and False alert rates using different thresholds for classification for alerts raised 1 (violet), 2 (light violet), 3 (dark pink) and 4 (green) weeks ahead. The black curve depicts the overall True and False alert rates. On each curve, the dot shows the True and False Alert rates at 42.5% threshold. For a given threshold (*x*th percentile of the forecast interval), we defined a True alert for a week where the *x*th percentile of the forecast interval and the observed incidence for a country were both greater than 0; false alert for a week where the threshold for a country was greater than 0 but the observed incidence for that country was 0; and missed alert for a week where the threshold for a country was 0 but the observed incidence for that country was greater than 0. True alert rate is the ratio of correctly classified true alerts to the total number of true and missed alerts (i.e., (true alerts)/(true alerts + missed alerts)). False alert rate is similarly the ratio of false alerts to the total number of false alerts and weeks of no alert (where the observed and the threshold incidence are both 0). **b** The True (green), False (orange) and Missed (red) 1 week ahead alerts using the 42.5th percentile of the forecast interval as threshold. The figure only shows countries on the African continent for which either the 42.5th percentile of the predicted incidence or the observed incidence was greater than 0 at least once. The first alert in each country is shown using larger squares. Alerts in a country in a week where there were no observed cases in the previous week are shown using triangles. In each case, weeks for which all observed points were imputed are shown in lighter shades. Country codes, shown on the y-axis, are as follows: CIV Côte d'Ivoire, GHA Ghana, GIN Guinea, LBR Liberia, MLI Mali, NGA Nigeria, SEN Senegal, SLE Sierra Leone, BFA Burkina Faso. The alerts are based on forecasts using ProMED data, a 2-week calibration window and a 4 week forecast horizon.

We found that the model allowed us to robustly predict the presence or absence of cases in all countries up to a week in advance. Overall, our model achieved high sensitivity (i.e., true alert rate) but variable specificity (i.e., 1 - false alert rate). Maximising the average between sensitivity and specificity led to 93.7% sensitivity and 96.0% specificity at 42.5% threshold (Fig. 2a). The sensitivity of the model remained high over a longer forecast horizon while the specificity deteriorated with more false alerts being raised 4 weeks ahead (Supplementary Fig. 47).

We tested the performance of the model in predicting the presence of cases in countries other than the three majorly affected countries (Guinea, Liberia and Sierra Leone). Both the sensitivity and the specificity of the model remained high under this more stringent criterion. The average of sensitivity and specificity was maximum at 92.5% threshold in this case with 85.7% sensitivity and 81.7% specificity (Supplementary Fig. 48).

Similarly, the model exhibited high sensitivity (83.3%) and specificity (82.0%) in predicting presence of cases in weeks following a week with no observed cases in all countries in Africa (Supplementary Fig. 49) at 93% threshold (chosen to maximise the average between sensitivity and specificity). Out of the 9 one-week ahead missed alerts in this case, 3 were in Liberia towards the end of the epidemic, after Liberia had been declared Ebola free on two separate occasions^27,28^. The serial interval distribution that we have used does not account for very long intervals between infections such as that associated with sexual transmission of Ebola Virus Disease^29^. Using the latest available data on pairs of primary and secondary infections and models that allow for more heterogeneity in the distribution of cases e.g., using Negative Binomial distributions could potentially improve the assessment of risk of spread in such cases^30,31^.

The choice of a threshold at which to raise an alert is subjective and involves a trade-off between sensitivity and specificity. In general, using a high threshold to raise an alert leads to high sensitivity with a reasonably high specificity (Supplementary Fig. 50). The cut-off for raising an alert can be informed by the relative costs of missed and false alerts potentially using a higher cut-off when the observed incidence is low. It is also worth noting that where our model failed to raise an alert in a week, either a true or a false alert had been raised in the recent weeks in all but one instance, indicating a potential risk of spread of the epidemic to that country.

These results suggest that the model is able to capture and even anticipate the spatial spread of the epidemic. Importantly, as the model is fitted to data from any of the three data sources accumulating over the course of the epidemic, it is able to predict the presence of cases relatively early in the epidemic (Supplementary Fig. 51) when such inputs would be particularly useful.

The risk of spatial spread in our model relies on estimating movement patterns of infectious cases. Our method also provides estimates of the probability of cases staying within a country throughout their infectious period, and the extent to which distance between two locations affects the flow of infectious cases between them. The real-time estimates of these parameters over the course of the epidemic (Supplementary Fig. 34), suggest that while the relative flow of cases between locations did not vary substantially over time, the probability of travel across national borders may have decreased after the initial phase of the epidemic.

**Fig. 3:**
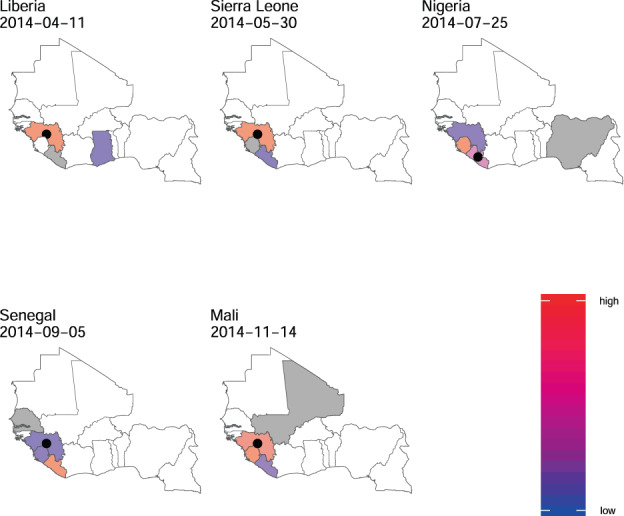
Relative risk of importation of the epidemic. For each country with non-zero incidence, the figure shows the relative importation risk (see Methods). Since we forecasted every 7th day, the risk of importation was estimated using forecasts closest to and before the date of the first case in that country reported in the data used. The date on which risk was estimated for each country is shown in the figure. Blue indicates low relative risk while deeper shades of red represent higher relative risk of acting as a source of importation. White is used to denote no risk. The estimates presented here use ProMED data with a 2 week calibration window. The country for which risk is estimated is shown in grey. The black circle represents the actual source of importation as retrospectively identified through epidemiological and genomic investigations. For each country, the figure shows only the risk of importation from other countries and does not show the risk of transmission within the country.

Finally, we quantify the relative risk of importation of a case into a country from any other currently affected country. The risk of importation is proportional to the population flow into a country from all other countries estimated using a mobility model (here, gravity model) weighted by the infectivity at each country (which depends on the number of cases and time at which they were infected, see Methods). Our estimates of the countries with higher risk of acting as source of importations were largely consistent with the reported source of cases (Fig. 3). In 4 out of the 5 reported cases of international spread of the epidemic in West Africa, the model correctly attributed the highest relative risk of acting as a source of importation (relative risk > 0.9 for importation into Liberia, Nigeria and Sierra Leone and 0.49 in case of Mali) to the actual source identified through retrospective epidemiological and genomic investigations^32^.

#### Short-term forecasts

**Fig. 4:**
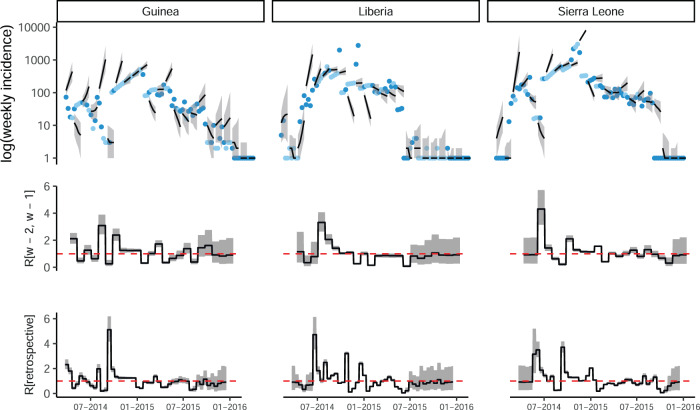
Observed and predicted incidence, and reproduction number estimates from ProMED data. **a** The weekly incidence derived from ProMED data and the 4 weeks incidence forecast on log scale. The solid dots represent the observed weekly incidence where the light blue dots show weeks for which all data points were obtained using interpolation. The projections are made over 4 week windows, based on the reproduction number estimated in the previous 2 weeks. **b** The reproduction number used to make forecasts over each 4 week forecast horizon. **c** The effective reproduction number estimated retrospectively using the full dataset up to the length of one calibration window before the end. In each case, the solid black line is the median estimate and the shaded region represents the 95% Credible Interval. The red horizontal dashed line indicates the *R*^*t*^ = 1 threshold. Results are shown for the three mainly affected countries although the analysis was done jointly using data for all countries in Africa.

**Fig. 5:**
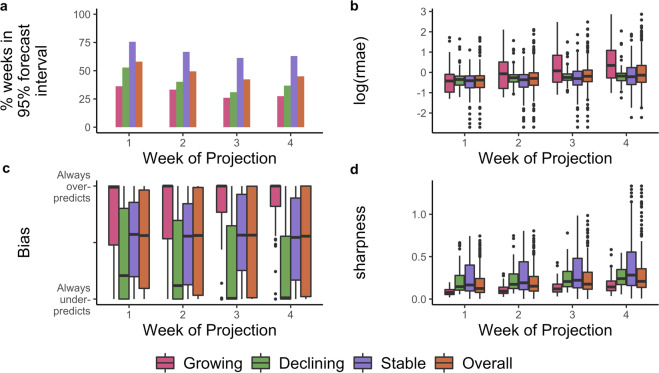
Model performance metrics overall and stratified by week of projection and epidemic phase. The performance metrics are **a** the percentage of weeks for which the 95% forecast interval contained the observed incidence, **b** relative mean absolute error, **c** bias, and **d** sharpness. In each panel, the bounds of the box represent the 25th and 75th percentiles and the line corresponds to the median of the distribution of the respective metric. The whiskers extend to 1.5 * Inter-quartile range in both directions. In forecasting ahead, we assumed transmissibility to be constant over the forecast horizon. If the 97.5th percentile of the R estimate used for forecasting was less than 1, we defined the epidemic to be in the declining phase during this period. Similarly, if the 2.5th percentile of R was greater than 1, we defined the epidemic to be in a growing phase. The phase was set to stable where the 95% Credible Interval of the R estimates contained 1. See Methods for definitions of each metric.

The ability of the model to robustly predict the future outbreak trajectory was limited and depended on the data source (Fig. 4) as well as on the time window used for inference (calibration window) and the forecast horizon. Results using a 2-week calibration window and a 4-week forecast horizon using ProMED data are presented in the main text (see Supplementary Figs. 6 and 7 for other forecast horizons and calibration windows). Overall, 48.7% of weekly observed incidence across all three countries were included in the 95% forecast interval (49.8% and 53.7% for HealthMap and WHO, respectively, Supplementary Table 1). Typically, model forecasts were 0.5 times lower or higher than the observed incidence (95% CrI 0.0–32.0) based on the relative mean absolute error (Fig. 5d), see Methods for details. We found no evidence of systematic bias in any week of the forecast horizon (median bias 0.12, Fig. 5a).

Typically, individual forecasts were within 17.0% (95% CrI 0–80%) of the median forecast (based on the median and 95% CrI for relative sharpness, Fig. 5b, see Methods).

As expected, the robustness of forecasts (both accuracy and precision) decreased over the forecast horizon (Fig. 5c). In the first week of the forecast window, 58.0% of observed values (across the three countries) were within the 95% forecast interval, reducing to 49.4%, 42.3% and 45.0% in the second, third and fourth weeks of the forecast horizon.

The model performance varied depending on the country and the phase of the epidemic, defined as "growing”, "declining”, and "stable” depending on *R*^*t*^ estimates (see phase definitions in Methods). In general, the model performance was best in the stable phase with 66.7% of the observations contained in the 95% forecasts interval (versus 40.2% and 30.8% in the declining and growing phases, respectively, Supplementary Table 1). However the forecast uncertainty was largest in the stable phase and smallest in the growing phase (Fig. 5b). Importantly, in the growing phase the model tended to over-predict while under-predicting in the declining phase (Fig. 5a).

Overall, the model performed moderately better using WHO data compared to ProMED and HealthMap data (Supplementary Fig. 33) and with shorter calibration windows (Supplementary Fig. 32).

Together with providing operational outputs such as the predicted short-term incidence in currently affected countries or the risk of spread to neighbouring countries, our method also provides near real-time estimates of parameters underlying the transmission model. First, the reproduction number *R*^*t*^ for each affected country is estimated over the time-window of inference, here over the most recent two weeks (Fig. 4), second row). We found that these near real-time estimates of *R*^*t*^ were in good agreement with retrospective estimates obtained using the entire incidence time-series (Fig. 4, bottom row, correlation coefficients varying between 0.58 and 0.90, Supplementary Fig. 5).

## Discussion

The rapid spread of COVID-19 from a province in China to more than 93 regions and countries around the world has brought into renewed focus the need for innovative strategies for epidemic monitoring. In this study, we propose a statistical framework that relies on digital surveillance data from ProMED or HealthMap to (1) quantify the short-term risk of spread to other countries, (2) for countries at risk of importation, quantify where the risk comes from, and (3) predict the short-term epidemic trajectory in currently affected countries. We applied our model to data collected during the West African Ebola epidemic of 2013–2016, curated by the outbreak analysts at ProMED/HealthMap, and we compared the model’s outputs to those obtained when using the data collated by the WHO and made available at the end of the epidemic.

In spite of the manual curation of the data carried out by outbreak analysts at ProMED and HealthMap, substantial issues remained in the quality and consistency of the data feeds. Dealing with issues such as missing data and inconsistent records will be a key challenge in using these data for prospective real-time analysis. Despite these challenges, we show the potential of digital surveillance tools to (1) inform early detection, (2) characterise the spread, and (3) forecast the future trajectory of outbreaks. Particularly in an evolving outbreak scenario, when information from traditional surveillance is limited and only available with a significant delay, digital surveillance data can be used to complement the information gap. For instance, during the West African Ebola epidemic, the first situation report by the WHO was published only at the end of August 2014^33^, reporting on cases between January and August 2014. On the other hand, the first post on ProMED on Ebola cases in Guinea appeared in March 2014^34^. Development of tools that can directly be plugged into the current digital surveillance ecosystem should therefore be a growing area of focus. This is further exemplified in the ongoing outbreak of COVID-19 where substantial efforts have been made by research groups around the world to collect, process and use publicly available data to calibrate models informing various aspects of the epidemiology of COVID-19^35–37^.

In general, we found that after systematic processing to remove inconsistencies, data from ProMED and HealthMap were in reasonably good agreement with the data collected through traditional surveillance methods and collated by WHO. There may be multiple reasons underpinning the observed discrepancies between ProMED and WHO data, including potential variability in digital surveillance reporting during the course of the epidemic. It is also worth highlighting that the WHO data used here are an extensively cleaned version of the data collected during the epidemic^38,39^, published more than one year after the epidemic was declared to be over. Moreover, while ProMED and HealthMap did not record probable cases for this epidemic, the WHO data contained confirmed, probable and suspected cases. As news media reports constitute one of the sources from which both ProMED/HealthMap extract case numbers, biases in reporting over the course of an outbreak are likely to have contributed to the data quality and completeness issues. Despite these discrepancies, the retrospective national estimates of transmissibility over time were well correlated across the three data sources. Further, estimates of transmissibility are typically robust to constant or slowly changing under-reporting. This suggests that digital surveillance data are a promising avenue for quantitative assessment of outbreak dynamics in real-time.

We used the ProMED/HealthMap data to perform a spatio-temporal analysis of the spread of the West African Ebola epidemic. We fitted a spatially explicit branching process model to the daily incidence data derived from ProMED/HealthMap feeds. The estimated model parameters were used to simulate the future outbreak trajectory over 4, 6 or 8 weeks. The model performs relatively well at short forecast horizon, i.e. up to two weeks. At a longer time scale, the model performance starts to deteriorate. A likely explanation for this is that our model assumes that transmissibility remains constant over the entire projection window. This assumption may not hold as we project over longer horizons, for example due to interventions being implemented. Model performance was also highly dependent on the phase of the epidemic in which projections were made. During the growing phase, the model tended to over-predict. This is likely due to interventions implemented throughout the growing phase to reduce transmissibility, leading to a reduced observed incidence compared to our model’s expectation. In the declining phase on the other hand, our model tended to under-predict the observed incidence. This could be due to control efforts being relaxed too early as case numbers decline. Another contributing factor could be super-spreading, a phenomenon observed in many epidemics including Ebola epidemics^40,41^ where a small number of cases generate a large number of secondary infections, implying that when case numbers are small, epidemic trajectory may be difficult to predict and not well described by Poisson likelihood. Models using more complex likelihoods, e.g. using Negative Binomial distributions, should be explored in future work, but will present additional challenges as analytical results underpinning the estimation of the reproduction number will no longer hold^26^.

Such variability in model performance throughout an epidemic could have important implications if the model predictions are used to inform resource allocation. Model estimates should therefore be interpreted with caution, particularly as an outbreak is observed to be declining, and if the forecast horizon is long.

We have shown that our model would have been able to accurately predict in real-time the international spread of Ebola in West Africa. Importantly, our model has very high sensitivity, predicting all instances of observed international spread 1–4 weeks in advance. Choosing a cut-off to maximise sensitivity led to low model specificity. On occasions the model predicted cases in countries, such as Côte d’Ivoire, where neither WHO nor ProMED reported any case. However this could be due to imperfect case reporting. Thus our method could be used with a high cut-off as a highly sensitive surveillance system with an alert triggering further epidemiological investigations and implementation of epidemic preparedness measures.

A key feature of our model is that it provides, for each country identified as at risk, a map of where the risk comes from. Out of 5 observed instances of international spread of Ebola in West Africa, our model correctly identified the source of importation in 4 cases while in the remaining case, the model highlighted the source of importation while assigning it low relative risk. This could help translating data collected through digital surveillance into concrete operational outputs in real-time that could assist in epidemic management and control.

The systematic collection, storage, organisation and communication of disease surveillance data were especially challenging during the West African Ebola epidemic as the deficiencies in transportation and communication resources, surveillance data quality and management, human resources and management structures posed unique challenges in this context^42^. The collection of case incidence data and rapid dissemination through digital surveillance systems was further hampered by the limited information technology and internet service in the countries most affected. Thus, for the West African Ebola epidemic, ProMED and HealthMap data were available at a coarse spatial scale with the sub-national level information for cases missing in most of the records. This limited our analysis to the spread of the outbreak across national borders only, although in principle our model could deal with data at any spatial scale. Both ProMED and HealthMap collect more data at a more granular geographic scale for most outbreaks. Utilising these data to investigate outbreaks and regions would provide further evidence of the ability of digital surveillance data to usefully complement data collected from traditional surveillance. Another interesting research avenue would be to explore ways of integrating timely data from ProMED and HealthMap with delayed data from ground surveillance to generate timely insights into the spatial spread of an outbreak.

Despite its known limitations^43^, and its application in this work to large spatial unit (countries), the gravity model of human movement is shown here to perform well in capturing the spread of the epidemic, albeit at coarse spatial scale. A related limitation of our approach is the assumption that the probability that a case will stay within a country during their infectious period is the same across all countries. In the absence of explicit mobility data, our model did not incorporate potentially non-uniform effects of the origin and destination population sizes on the flow of people. The availability of fine-grained mobility data would allow us to perform similar analyses at a more resolved spatial scale, and calibrate the model when restrictive assumptions are relaxed. Mobile phone usage data have been used to infer human mobility patterns to model the spread of diseases^44–48^. Despite the potential biases in these data, they can be immensely useful in understanding the patterns of human mobility at a spatial and temporal scale relevant to the spread of infectious diseases outbreaks. Other data sources such data on the number of flights and/or passengers across countries, or location data from social media have been used to model the spread of outbreaks^49–51^. However these data are neither free nor easy to acquire and their utility in near real-time outbreak analytics remains challenging^52,53^. In limited instances, where organisations have made aggregated data on human population movement available for research, these have been used to answer key questions about the spread of the ongoing COVID-19 pandemic^54,55^.

The framework presented in this paper was developed as a proof-of-concept to use digital surveillance data for near real-time forecasting of the spatio-temporal spread of an outbreak. It has been implemented as a web-based tool called "Mapping the Risk of International Infectious Disease Spread” (MRIIDS) (see^56^ for more information). To further develop such approaches, it is important to establish an automated pipeline from data collection to curation and analysis, which currently requires manual intervention at each of these steps. Although the computational time needed for data-preprocessing was negligible (in the order of a few seconds to a minute), extraction of data from a ProMED report can take from 3 to 10 min, depending on the complexity of the report. Ongoing efforts to automate the extraction of quantitative information from ProMED and HealthMap^57^ will further enhance the utility of these tools for real-time outbreak analysis. Another factor that could improve the usability of our model in near real time is to reduce the running time of the fitting and forward simulation. In the current implementation, the running time varies from ~0.5 CPU hours when 100 days of incidence data are being used to ~335 CPU hours using 462 days of incidence data using a 3.3 GHz Intel Xeon X5680 processor. Although the West African Ebola epidemic was of unusual scale and duration, there is a scope for optimising the model implementation.

Importantly, many other open data sources could be included in our framework to improve model performance. For example, data on human mobility could be used to further inform the parametric form and parameter values of our mobility model. We have incorporated a simple and well-characterised model of population movement in the current work. In addition to utilising other possible data sources, future work could consider other models of human population movement^43^. When relevant, spatially explicit data on population-level immunity to the circulating pathogen (e.g. following previous epidemics and/or due to vaccination) could also be used to refine our transmission model. The effective reproduction number ${R}_{i}^{t}$ is affected by a number of factors e.g., the intrinsic transmissibility of a pathogen or the health care capacity at location *i* (which could influence for example the capacity to isolate cases). The model could be easily extended to explicitly incorporate the dependence of the reproduction number on such factors. Finally, the impact of the health capacity of a region to respond to a public health emergency could also be accounted for in future iterations of the model.

Ongoing efforts to collate quantitative information on the performance of health systems and the ability of regions or countries to respond to an epidemic^58,59^ can potentially provide valuable data for future work. Here using a relatively simple modelling approach we provide one of the first pieces of evidence of the potential value of digital surveillance for real-time quantitative analysis of epidemic data, with important operational and actionable outputs.

## Methods

### Case incidence data

We used a set of curated ProMED and HealthMap records on the human cases of Ebola Virus Disease in West Africa. The dates in the feeds ranged from March 2014 to October 2016. Each dataset recorded the cumulative number of suspected/confirmed cases and suspected/confirmed deaths by country at various dates in this period. We derived the country specific daily and weekly incidence time series from these data after data cleaning and pre-processing steps that consisted of removing duplicate and/or inconsistent points, and interpolating the missing points (see Section 1). The raw and processed data from ProMED and HealthMap for all countries included in the feed are available in the Github repository for this article.

We also used the West African Ebola incidence data collated by the WHO during the epidemic which was made available ~1 year after the end of the epidemic^60^. This data set is referred to as "WHO data” in the interest of brevity. The cleaned version of the WHO data consisted of cases reported between December 2013 and October 2015 in the three most affected countries - Guinea, Sierra Leone and Liberia. The location of residence of cases was geo-coded to the second administrative level. We aggregated the WHO data to national level to match the spatial resolution of ProMED and HealthMap data that were only available at the country level.

Since the data collected by ProMED and HealthMap were manually curated by outbreak analysts, we have used the word "curate” in referring to their data collection process. Similarly, we refer to the data "collated” by WHO as WHO coordinated the international response to the outbreak and in this role, they collated the information from Ministries of Health, situation reports from NGOs, and local and international medical teams.

As HealthMap uses ProMED alerts in addition to other online data sources, ProMED represents the more conservative data source between the two. Therefore we present the results based on ProMED data in the main text, unless otherwise specified. The analysis based on HealthMap and WHO data are presented in the Supplementary Information (Sections 3 and 4).

### Demographic data

We used LandScan^TM^ 2015 dataset grid^61^ for population estimates and centroids for all countries on African mainland. The maps were created using shapefiles from the GADM database version 3.6 (https://gadm.org).

### Statistical model

Our model relies on a well-established statistical framework that assumes the daily incidence, *I*_*t*_, can be approximated with a Poisson process following the renewal equation^25^:

1 $${I}_{t} \sim Pois\left({R}^{t}\mathop{\sum }\limits_{s=1}^{t}{I}_{i}^{t-s}{\omega }_{s}\right).$$

Here *R*^*t*^ is the reproduction number at time *t* (the average number of secondary cases per infected individual) and *ω* is the distribution of the serial interval (the time between onset of symptoms in a case and their infector).

We extend this model to incorporate the spatial spread of the outbreak between *n* different locations. We assume that the number of incident cases at a location *j* at time *t* is given by the equation

2 $${I}_{j}^{t} \sim Pois\left({{{\Lambda }}}_{j}^{t}\right),$$

where ${{{\Lambda }}}_{j}^{t}$ is the total infectivity at a location *j* at time *t*. ${{{\Lambda }}}_{j}^{t}$ is the sum of infectivity at all locations weighted by the relative flow of cases into *j* from each location *i*. That is,

3 $${{{\Lambda }}}_{j}^{t}=\mathop{\sum }\limits_{i=1}^{n}\left({p}_{i\to j}{R}_{i}^{t}\mathop{\sum }\limits_{s=1}^{t}{I}_{i}^{t-s}{\omega }_{s}\right).$$

${R}_{i}^{t}$ is the reproduction number at location *i* at time *t* and *p*_*i*→*j*_ is the probability of a case moving from location *i* to location *j* while they are infectious. *ω* is the typical infectiousness profile of a case over time after infection.

The probability of moving between locations is derived from the relative flow of populations between locations. This latter quantity can be estimated using a population flow model; here we use a gravity model^62,63^. Under a gravity model, the flow of individuals from area *i* to area *j*, *ϕ*_*i*→*j*_, is proportional to the product of the populations of the two locations, *N*_*i*_ and *N*_*j*_ and inversely proportional to the distance between them *d*_*i*,*j*_ raised to a power *γ*:

4 $${\phi }_{i\to j}=\frac{{N}_{i}{N}_{j}}{{d}_{i,j}^{\gamma }}.$$

The exponent *γ* modulates the effect of distance on the flow of populations. A large value of *γ* indicates that the distances travelled by populations tend to be short. The probability of movement from location *i* to location *j* where (*j* ≠ *i*) is then given by

5 $${p}_{i\to j}=(1-{p}_{stay})\frac{{\phi }_{i\to j}}{\sum _{x,x\ne i}{\phi }_{i\to x}},$$

where *p*_*stay*_ is the probability that a case remains in a location *i* throughout their infectious period i.e. *p*_*stay*_ is *p*_*i*→*i*_.

### Short-term forecasts

To forecast the number of cases and estimate the risk of spatial spread at time *t*, we fitted a spatially explicit branching process model to the daily incidence in all locations up to *t* − 1. We assumed a single *p*_*stay*_ for all countries on African mainland. For estimating the time-varying reproduction number for each country, we split the duration of the total outbreak into intervals of equal width (calibration time window). We assume that transmissibility in each location stays constant within each time window and thus, within a time window, we estimated a single reproduction number for each country with non-zero incidence. We varied the length of the time window to obtain different models, with short time windows increasing the number of parameters in the model. We used the estimates of the effective reproduction number in the last time window to forecast ahead and assumed that transmissibility remains constant throughout the forecast horizon.

The results presented in the main text use a calibration time window of 2 weeks and a forecast horizon of 4 weeks (see Supplementary Sections 2.1 and 2.3 for other results).

### Relative risk of importation

Let ${{{\Lambda }}}_{j}^{t}$ denote the infectivity at a location *j* at time *t* (Eq. (3)) as before. We define the risk of importation of a case from *i* into *j* as the proportion of total infectivity at *j* at time *t*, ${{{\Lambda }}}_{j}^{t}$, due to infectivity at *i*. That is,

6 $${r}_{j\leftarrow i}^{import}(t)=\frac{{p}_{i\to j}{\lambda }_{i}^{t}}{{{{\Lambda }}}_{j}^{t}},$$

where ${{{\Lambda }}}_{j}^{t}$ is as in Eq. (3), and ${\lambda }_{i}^{t}={R}_{i}^{t}\mathop{\sum }\limits_{s=1}^{t}{I}_{i}^{t-s}{\omega }_{s}$.

### Transmissibility parameters

We assumed a Gamma distributed serial interval with mean 14.2 days and standard deviation 9.6 days^39^. For the reproduction number, we used a Gamma prior with mean 3.3 and variance 1.5. This was informed by a review of estimates of the reproduction number for Ebola Zaire in outbreaks preceding the West African Ebola outbreak which reported estimates ranging from 1.4 to 4.7^64^. The mean prior 3.3 was chosen as the midpoint of this interval, and the variance 1.5, was chosen so the 95% prior probability interval contains the extremes of this interval.

### Gravity model parameters

For the gravity model parameters *p*_*stay*_ and *γ*, we chose uninformative uniform priors to allow population movement to be informed by the epidemiological data. Since *p*_*stay*_ is a probability, the prior was a uniform distribution on the interval [0, 1]. *γ* was allowed to vary between 1 and 2 in the results presented in the main text. We performed a sensitivity analysis where *γ* has a uniform prior between 1 and 10. The results of this analysis are presented in the Supplementary Section 8.

### Model fitting

To reduce the number of parameters in the model, we divided the countries with non-zero incidence into 5 groups and forced each country in a group to have the same reproduction number in each time window. The first three groups consist of the three mainly affected countries - Sierra Leone, Guinea and Liberia. The countries that shared a border with these three countries were grouped together. These were Mali, Côte d’Ivoire, Guinea-Bissau and Gambia. The rest of the countries were assigned to the fifth group. A comparison of the performance of different models is presented in the Supplementary Material (Supplementary Fig. 32).

Model fitting was done in a Bayesian framework using Markov Chain Monte Carlo (MCMC) as implemented in the software Stan^65^ and its R interface rstan^66^. We ran 2 MCMC chains with 3000 iterations and burn-in of 1000 iterations. Convergence of MCMC chains was confirmed using visual inspections of the diagnostics (Potential Scale Reduction Factor^67^ and Geweke Diagnostics^68^) reported by R package ggmcmc^69^. An example report produced by ggmcmc is included in the Supplementary Material.

For each model (i.e., for each choice of the time window), we made forward projections every 7th day, over a 2 week, 4 week and 6 week horizon. To forecast incidence from day *t* onwards, we fitted the model to the daily incidence series up to day *t* − 1. We then sampled 1000 parameter sets (reproduction numbers for each location in each time window and parameters of the gravity model) from the joint posterior distribution, and for each parameter set, simulated one future epidemic trajectory according to equation (1), assuming that future *R*_*t*_ is equal to the last estimated *R*_*t*_ value in each location.

### Model validation

Given the retrospective nature of our analysis, we validated the incidence projected using our model against observed incidence, using the data source which was used to fit the model. That is, when the model was fitted to ProMED (respectively HealthMap/WHO) data, the projected incidence was compared with the observed incidence in the ProMED (respectively HealthMap/WHO) data. When comparing the model performance across the three data sources, we restricted model outputs to the dates for which data from all three sources was available (12th April 2014 to 2nd January 2016, from ProMED data).

In addition to the accuracy of the forecasted incidence, the uncertainty associated with the forecasts (e.g., measured by the width of the prediction interval) is an important indicator of model performance. A narrow prediction interval that contains the observed values is preferable over wide prediction intervals. To assess the performance of the model along both these dimensions, we used four different metrics drawn from the literature.

In the remainder of this paper, we use the following notation. For a location *j*, let ${I}_{j}^{t}$ be the observed incidence at time *t* and let ${\hat{I}}_{j}^{t}$ be the set of predictions of the model at time *t*. That is, ${\hat{I}}_{j}^{t}=\{{\hat{I}}_{j}^{t,1},{\hat{I}}_{j}^{t,2},\ldots {\hat{I}}_{j}^{t,N}\}$ is the set of *N* draws from the Poisson distribution with mean ${{{\Lambda }}}_{j}^{t}$ (Equation 1) (here *N* = 1000).

#### Relative mean absolute error

The relative mean absolute error (rmae) is a widely used measure of model accuracy^70^. The relative mean absolute error for the forecasts at a location *j* at time *t* is defined as:

7 $$rma{e}_{j}^{t}({I}_{j}^{t},{\hat{I}}_{j}^{t})=\frac{\mathop{\sum }\nolimits_{s = 1}^{N}| {I}_{j}^{t}-{\hat{I}}_{j}^{t,s}| }{N* ({I}_{j}^{t}+1)}.$$

That is the mean absolute error at time *t* is averaged across all simulated incidence trajectories and normalised by the observed incidence. We add 1 to the observed value to prevent division by 0. A rmae value of *k* means that the average error is *k* times the observed value.

#### Sharpness

Sharpness is a measure of the spread (or uncertainty) of the forecasts. Adapting the definition proposed by Funk et al.^71^, we used the relative mean deviation about the median to evaluate sharpness. The sharpness ${s}_{j}^{t}$ of forecasts at time *t* at location *j* is

8 $${s}_{j}^{t}({\hat{I}}_{j}^{t})=mean\left(\frac{| {\hat{I}}_{j}^{t,s}-median({\hat{I}}_{j}^{t})| }{median\left({\hat{I}}_{j}^{t}+1\right)}\right).$$

We add 1 to ${\hat{I}}_{j}^{t}$ to prevent division by 0. A sharpness score of *k* indicates that the average deviation of the predicted incidence trajectories is *k* times their median. Low values of sharpness therefore suggest that the predicted trajectories are clustered around the median.

#### Bias

The bias of forecasts is a measure of the tendency of a model to systematically under- or over-predict^71^. The bias of a set of predictions ${\hat{I}}_{j}^{t}$ at time *t* at location *j* is defined as

9 $${b}_{j}^{t}({I}_{j}^{t},{\hat{I}}_{j}^{t})=2\left(mean\left(H\left({\hat{I}}_{j}^{t,s}-{I}_{j}^{t}\right)\right)-0.5\right),$$

where the mean is taken across the *N* draws. *H*(*x*) is the Heaviside step function defined as

10 $$H(x)=\left\{\begin{array}{ll}0&\,\text{if}\,\ x\;<\;0\\ 1&\,\text{if}\,\ x\;>\;0\\ 0.5&\,\text{if}\,\ x=0.\end{array}\right.$$

The above formulation can better be understood by considering the following extreme scenarios. If every projected value ${\hat{I}}_{j}^{t}$ is greater than the observed value ${I}_{j}^{t}$, then the Heaviside function is 1 for all *i* = 1, 2, …*N*, and $mean\left(H\left({\hat{I}}_{j}^{t}-{I}_{j}^{t}\right)\right)$ is 1. The bias for a model that always over-predicts is therefore 1. On the other hand, if the model systematically under-predicts, then $mean\left(H\left({\hat{I}}_{j}^{t}-{I}_{j}^{t}\right)\right)$ is 0 and the bias is -1. For a model for which all predictions match the observed values exactly, the bias is 0.

### Epidemic phase

We defined the epidemic to be in a "growing" phase at time *t* if the 2.5th percentile of the distribution of the reproduction number at this time was greater than 1. Similarly, the epidemic was defined to be in "declining" phase if the 97.5th percentile of the distribution of the reproduction number was below 1. In all other cases, the epidemic was defined to be in a "stable" phase.

### Reporting summary

Further information on research design is available in the Nature Research Reporting Summary linked to this article.

## Supplementary information

Supplementary Information

Reporting Summary

## Data Availability

The raw data from ProMED and HealthMap are available at https://github.com/sangeetabhatia03/mriids_manuscript/tree/master/data/raw. The demographic data (population and centroids) used are available at https://github.com/sangeetabhatia03/mriids_manuscript/tree/master/data/processed. The code for processing ProMED and HealthMap data are available at https://github.com/sangeetabhatia03/mriids_manuscript.

## Code availability

All analysis was carried out in the statistical software R version 3.5.3. The code for analysis of ProMED and HealthMap data and an implementation of the model is available online (https://github.com/sangeetabhatia03/mriids_manuscript).
