# Supplementary material for: Using digital surveillance tools for near real-time mapping of the risk of infectious disease spread
Source: NPJ Digit Med. 2021 Apr 16;4:73. doi: 10.1038/s41746-021-00442-3 (PMC8052406; doi:10.1038/s41746-021-00442-3)
Supplement: Supplementary file 1 — Supplementary Information [file 41746_2021_442_MOESM1_ESM.pdf]

Using Digital Surveillance Tools for Near Real-Time Mapping of the Risk of Infectious Disease Spread (Supplementary Information)

Sangeeta Bhatia[1], Britta Lassmann[2], Emily Cohn[3], Angel N. Desai[2], Malwina Carrion[2, 4], Moritz U. G. Kraemer[3, 5, 6], Mark Herringer[7], John Brownstein[3], Larry Madoff[2], Anne Cori[1], and Pierre Nouvellet[1, 8]

[1]MRC Centre for Global Infectious Disease Analysis, School of Public Health, Imperial College London, Faculty of Medicine, Norfolk Place, London, W2 1PG, UK
[2]ProMED, International Society for Infectious Diseases, Brookline, MA 02446 USA
[3]Computational Epidemiology Group, Division of Emergency Medicine, Boston Children's Hospital, Boston, MA USA
[4]Department of Health Science, Sargent College, Boston University, Boston, MA USA
[5]Department of Zoology, Tinbergen Building, Oxford University, Oxford, UK
[6]Department of Pediatrics, Harvard Medical School, Boston, USA
[7]healthsites.io, London, WC1A 1DG, UK
[8]School of Life Sciences, University of Sussex, Brighton, UK

March 16, 2021

Contents

1 Processing ProMED and HealthMap data 4
1.1 Data cleaning 4
1.2 Weekly incidence from ProMED, HealthMap and WHO data 7
1.3 Correlation between R estimates 7
2 Forecasts using ProMED data 10
2.1 Calibration window of 2 weeks 10
2.1.1 Forecast horizon of 4 weeks 10
2.1.2 Forecast horizon of 6 weeks 10
2.1.3 Forecast horizon of 8 weeks 12
2.2 Calibration window of 4 weeks 14
2.2.1 Forecast horizon of 4 weeks 14
2.2.2 Forecast horizon of 6 weeks 16
2.2.3 Forecast horizon of 8 weeks 18
2.3 Calibration window of 6 weeks 20
2.3.1 Forecast horizon of 4 weeks 20
2.3.2 Forecast horizon of 6 weeks 22
2.3.3 Forecast horizon of 8 weeks 24
3 Forecasts using HealthMap data 26
3.1 Calibration window of 2 weeks 26
3.1.1 Forecast horizon of 4 weeks 26
3.1.2 Forecast horizon of 6 weeks 28
3.1.3 Forecast horizon of 8 weeks 30
3.2 Calibration window of 4 weeks 32
3.2.1 Forecast horizon of 4 weeks 32
3.2.2 Forecast horizon of 6 weeks 34
3.2.3 Forecast horizon of 8 weeks 36
3.3 Calibration window of 6 weeks 38
3.3.1 Forecast horizon of 4 weeks 38
3.3.2 Forecast horizon of 6 weeks 40
3.3.3 Forecast horizon of 8 weeks 42
4 Forecasts using WHO data 44
4.1 Calibration window of 2 weeks 44
4.1.1 Forecast horizon of 4 weeks 44
4.1.2 Forecast horizon of 6 weeks 46
4.1.3 Forecast horizon of 8 weeks 48

4.2 Calibration window of 4 weeks 50
4.2.1 Forecast horizon of 4 weeks 50
4.2.2 Forecast horizon of 6 weeks 52
4.2.3 Forecast horizon of 8 weeks 54
4.3 Calibration window of 6 weeks 56
4.3.1 Forecast horizon of 4 weeks 56
4.3.2 Forecast horizon of 6 weeks 58
4.3.3 Forecast horizon of 8 weeks 60
5 Impact of calibration window on model performance 62
6 Impact of datasource on model performance 62
7 Mobility Model Parameters 64
8 Sensitivity Analysis 64
8.1 Forecasts using ProMED data 67
8.1.1 Calibration window of 2 weeks 67
8.1.2 Forecast horizon 4 weeks 67
8.1.3 Forecast horizon 6 weeks 69
8.1.4 Forecast horizon 8 weeks 69
8.1.5 Calibration window of 4 weeks 71
8.1.6 Forecast horizon 4 weeks 71
8.1.7 Forecast horizon 6 weeks 71
8.1.8 Forecast horizon 8 weeks 73
8.1.9 Calibration window of 6 weeks 73
8.1.10 Forecast horizon 4 weeks 73
8.1.11 Forecast horizon 6 weeks 75
8.1.12 Forecast horizon 8 weeks 75
8.2 Model performance with alternate priors for mobility model parameter 77
9 Risk of spatial spread 79
10 Model Convergence Diagnostic Report 84

Overview

In this supplement, we present the details of the pre-processing of ProMED and HealthMap feeds (Section 1), the model results using ProMED, HealthMap and WHO data and the impact of the datasources and model parameters on the performance of the model.

We varied the length of the time window used for model fitting (see Methods for details). Sections 2, 3 and 4 present the forecasts using ProMED, HealthMap and WHO data respectively using different calibration windows (2, 4 and 6 weeks) and forecast horizons (4, 6 and 8 weeks). The model performance was moderately better with shorter calibration windows (Supplementary Figure 32) and with WHO data (Supplementary Figure 33). We explored the impact of alternate priors for the gravity model parameter on the results from the model. The results of this sensitivity analysis are presented in Section 8. We present additional analysis on the predicting the spatial spread of the epidemic in Section 9.

1 Processing ProMED and HealthMap data

1.1 Data cleaning

The data on cumulative number of confirmed and suspected Ebola cases in Guinea, Liberia, Sierra Leone, Senegal, Mali, Nigeria and Ghana were extracted from ProMED and HealthMap feeds. We derived the country specific daily and weekly incidence time series from these data after the following data cleaning and pre-processing:

- We first extracted the total case counts as a sum of suspected and confirmed cases (ProMED and HealthMap data did not record probable cases for the West African Ebola epidemic).

- Each unique record in ProMED is associated with a unique alert id. An alert id can correspond to various reports from different sources (news, social media etc.) which might report different case numbers. In such cases, we assigned the median of the case numbers to the record.

- In some instances, cumulative case count was inconsistent in that it failed to be monotonically non-decreasing. We identified consecutive dates (t_k and t_{k+1}) where the cumulative case count was not increasing. If removing either t_k or t_{k+1} made the cumulative case count increasing, we adopted this option. If however removing both or none of them resulted in a increasing series, we removed both t_k and t_{k+1}. These rules were applied iteratively until the cumulative case count was consistent.

- If an inconsistent point was at the end of the the cumulative case series, applying the above rules led to the removal of a large number of points. Hence, to remove outliers at the end, we used Chebyshev inequality with sample mean [1]. Given a set of observations $X_1, X_2 \ldots X_n$, the formulation of Chebyshev inequality by Saw et al. gives the probability that the observation X_{n+1} is within given sample standard deviations of the sample mean. We defined X_{n+1} to be an outlier if the probability of observing this point given observations $X_1, X_2 \ldots X_n$ is less than 0.5. Fixing this probability allowed us to determine k such that $Pr(\mu - k\sigma \leq X\_\{n+1\} \leq \mu + k\sigma) \geq 0.5$, where μ and σ are the sample mean and sample standard deviation respectively. We deleted an observation X_{n+1} as an outlier if it did lie in this interval.

- Finally, cumulative incidence on days with no records was filled in using log-linear interpolation.

The pre-processing workflow is illustrated using data for Sierra Leone from ProMED.

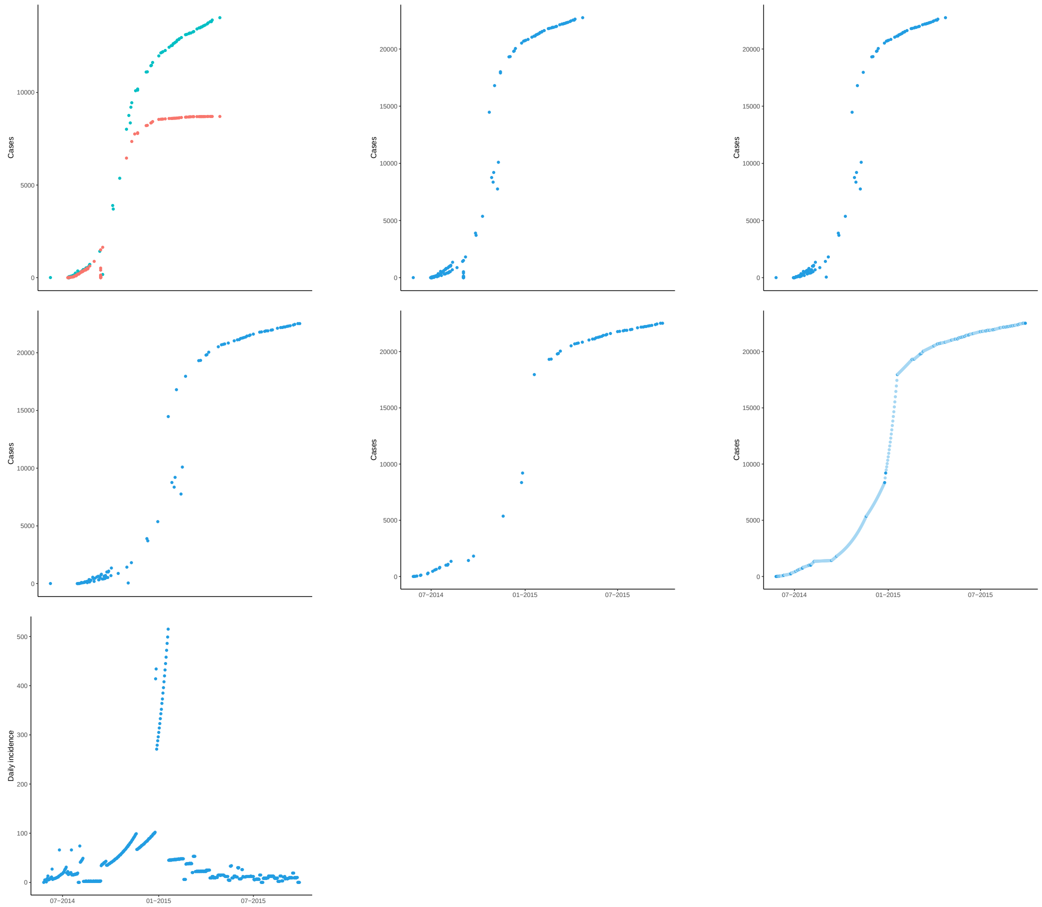

Supplementary Figure 1: Illustration of workflow for Processing ProMED feed. Raw ProMED feed consisted of suspected and confirmed cases and suspected and confirmed deaths. The top left figure (a) shows the suspected (teal) and confirmed (red) cases in Sierra Leone in the raw feed. We used the suspected and confirmed cases to derive cumulative incidence data (b). If there were more than one alert on a day, these were then removed by assigning the median of the cases reported to this day. (c). If there were outliers in the data, we removed them in the next step (d). We then made the cumulative case count monotonically increasing (e) by removing inconsistent records. Finally, missing data were imputed using log-linear interpolation (f). Imputed points are show in light blue. See Methods for details.

1.2 Weekly incidence from ProMED, HealthMap and WHO data

We processed the data from ProMED and HealthMap and derived daily and weekly incidence. The weekly incidence series derived from the three data sources were highly correlated (Supplementary Figure 2).

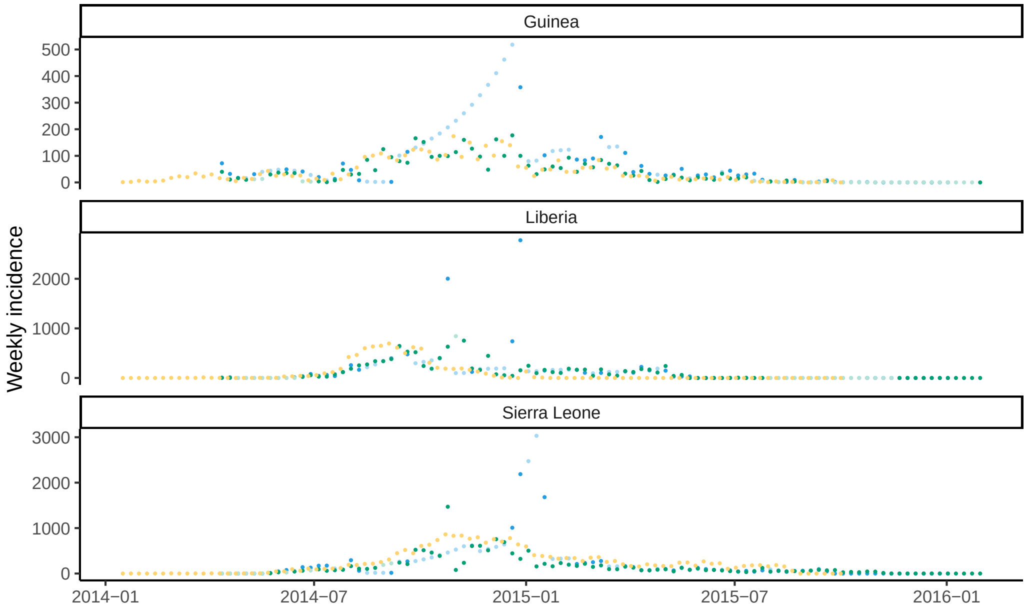

Supplementary Figure 2: Comparison of national weekly incidence trends from ProMED (blue), HealthMap (green) and WHO (yellow) data for Guinea, Liberia and Sierra Leone. Weeks for which all daily data points were imputed are shown in lighter shades of blue and green respectively. The y-axis differs for each plot.

1.3 Correlation between R estimates

The correlation between estimates of time-varying reproduction number estimated using ProMED, HealthMap and WHO data depended on the time window used for estimation and the country (Supplementary Figure 3). Restricting the analysis was robust to using reproduction number estimates with lower uncertainty (coefficient of variation less than 0.25, Supplementary Figure 4) Similarly, for each data source, the retrospective estimates

of reproduction number in the Bayesian framework using the full incidence curve were in reasonably good agreement with the real-time estimates. The strength of the correlation varied depending on the country and the length of the calibration window (Supplementary Figure 5).

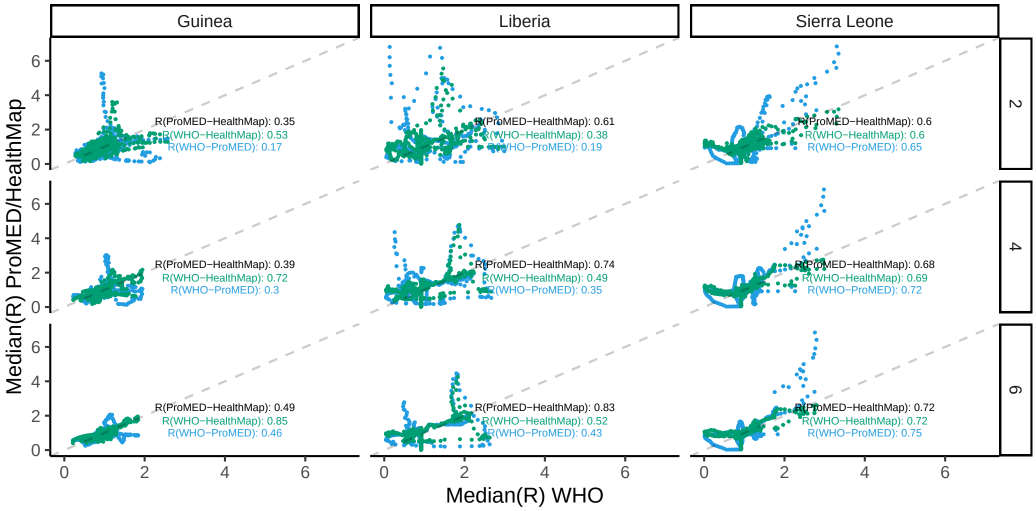

Supplementary Figure 3: Correlation between time-varying reproduction number estimated from ProMED, HealthMap and WHO data. The reproduction numbers were estimated using R package EpiEstim over a 4 week sliding window. Median estimates from WHO data are on the x-axis and the median estimates using ProMED (blue) and HealthMap (green) data are on the y-axis. All correlation coefficients were statistically significant.

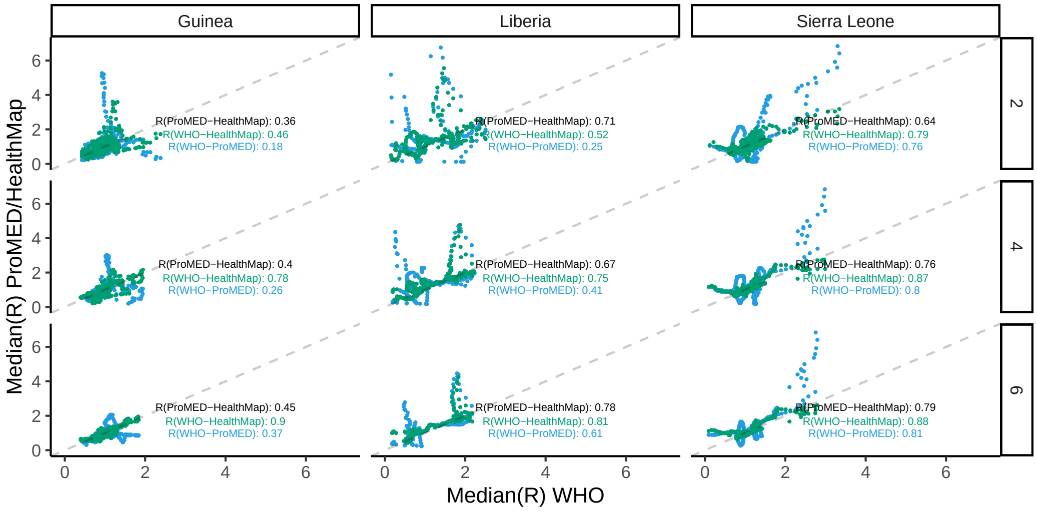

Supplementary Figure 4: Correlation between time-varying reproduction number estimated from ProMED, HealthMap and WHO data. Only estimates with a coefficient of variation less than 0.25 were included in this analysis. The reproduction numbers were estimated using R package EpiEstim over a 4 week sliding window. Median estimates from WHO data are on the x-axis and the median estimates using ProMED (blue) and HealthMap (green) data are on the y-axis. All correlation coefficients were statistically significant.

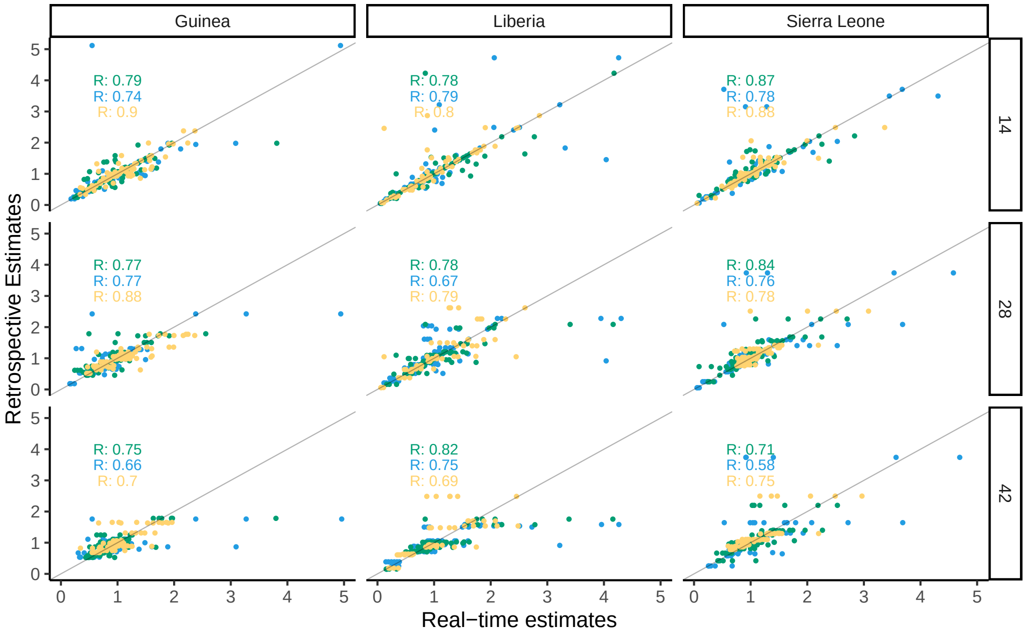

Supplementary Figure 5: Correlation between real-time and retrospective estimates of time-varying reproduction number from ProMED (blue), HealthMap (green) and WHO (yellow) data. Median real-time estimates are on the x-axis and the median retrospective estimates are on the y-axis. All correlation coefficients were statistically significant.

2 Forecasts using ProMED data

This section presents the forecast produced using ProMED data with calibration window of 2 (Section 2.1), 4 (Section 2.2) and 6 (Section 2.3) weeks over a forecast horizon of 4, 6 and 8 weeks. Results using calibration window of 2 weeks and forecast horizon of 4 weeks are presented in the main text.

2.1 Calibration window of 2 weeks

2.1.1 Forecast horizon of 4 weeks

2.1.2 Forecast horizon of 6 weeks

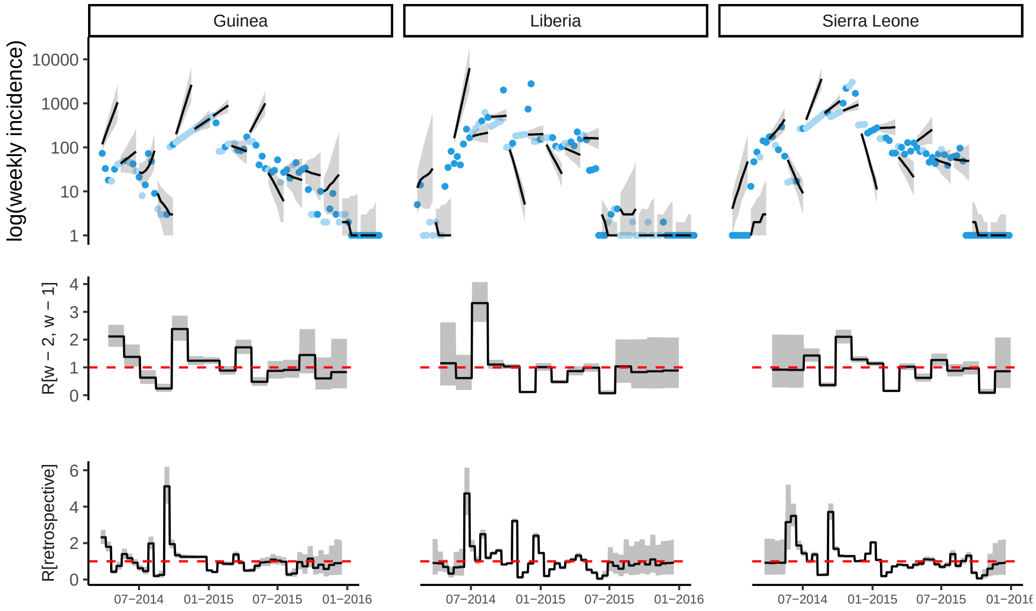

Supplementary Figure 6: Observed and predicted incidence, and reproduction number estimates from ProMED data. The top panel shows the weekly incidence derived from ProMED data and the 6 weeks incidence forecast on log scale. The solid dots represent the observed weekly incidence where the light blue dots show weeks for which all data points were obtained using interpolation. The projections are made over 6 week windows, based on the reproduction number estimated in the previous 2 weeks. The middle figure in each panel shows the reproduction number used to make forecasts over each 6 week forecast horizon. The bottom figure shows the effective reproduction number estimated retrospectively using the full dataset up to the length of one calibration window before the end. In each case, the solid black line is the median estimate and the shaded region represents the 95% Credible Interval. The red horizontal dashed line indicates the $R_t = 1$ threshold. Results are shown for the three mainly affected countries although the analysis was done jointly using data for all countries in Africa.

Supplementary Table 1: Percentage of weeks with observed incidence in 95% forecast interval. In forecasting ahead, we assumed transmissibility to be constant over the forecast horizon. If the 97.5th percentile of the R estimate used for forecasting was less than 1, we defined the epidemic to be in the declining phase during this period. Similarly, if the 2.5th percentile of R was greater than 1, we defined the epidemic to be in a growing phase. The phase was set to stable where the 95% Credible Interval of the R estimates contained 1.

Country	Growing	Declining	Stable	Overall
Guinea	44.0%	42.2%	71.9%	54.4%
Liberia	18.3%	31.5%	69.5%	49.3%
Sierra Leone	25.9%	46.0%	55.7%	42.5%
All	30.8%	40.2%	66.7%	48.7%

2.1.3 Forecast horizon of 8 weeks

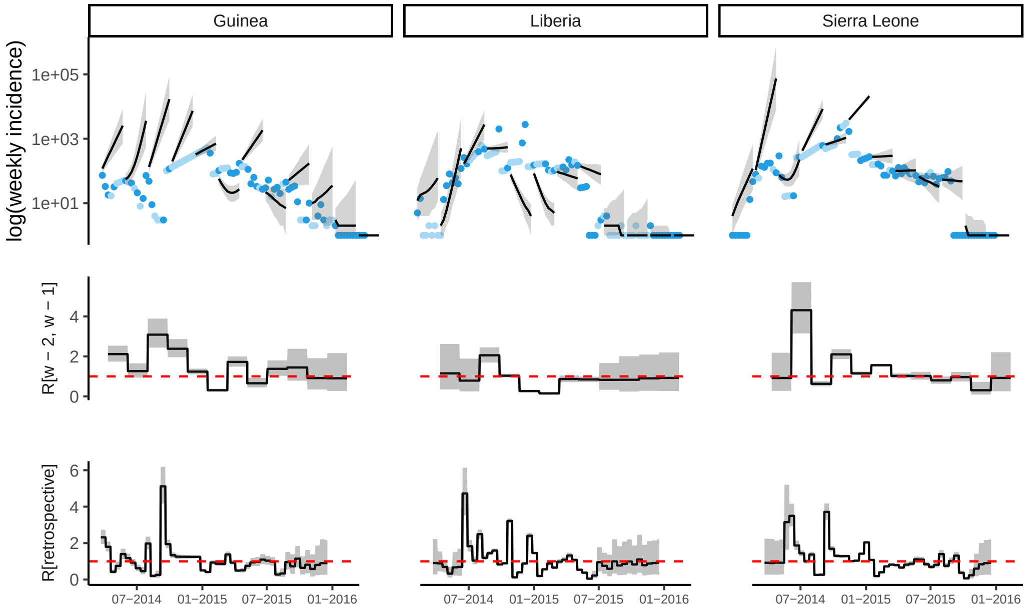

Supplementary Figure 7: Observed and predicted incidence, and reproduction number estimates from ProMED data. The top panel shows the weekly incidence derived from ProMED data and the 8 weeks incidence forecast on log scale. The solid dots represent the observed weekly incidence where the light blue dots show weeks for which all data points were obtained using interpolation. The projections are made over 8 week windows, based on the reproduction number estimated in the previous 2 weeks. The middle figure in each panel shows the reproduction number used to make forecasts over each 8 week forecast horizon. The bottom figure shows the effective reproduction number estimated retrospectively using the full dataset up to the length of one calibration window before the end. In each case, the solid black line is the median estimate and the shaded region represents the 95% Credible Interval. The red horizontal dashed line indicates the $R_t = 1$ threshold. Results are shown for the three mainly affected countries although the analysis was done jointly using data for all countries in Africa.

2.2 Calibration window of 4 weeks

2.2.1 Forecast horizon of 4 weeks

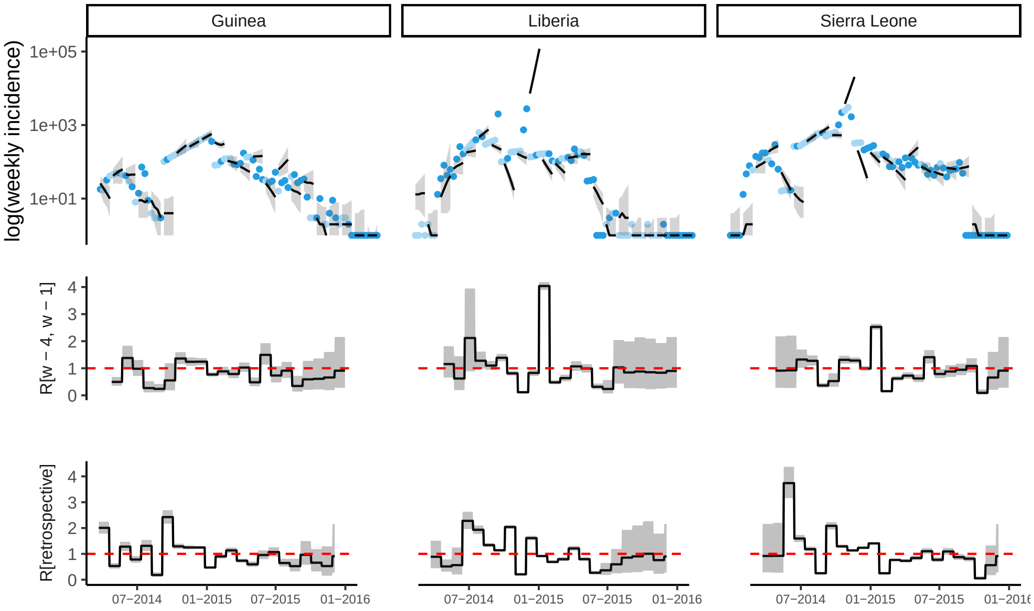

Supplementary Figure 8: Observed and predicted incidence, and reproduction number estimates from ProMED data. The top panel shows the weekly incidence derived from ProMED data and the 4 weeks incidence forecast on log scale. The solid dots represent the observed weekly incidence where the light blue dots show weeks for which all data points were obtained using interpolation. The projections are made over 4 week windows, based on the reproduction number estimated in the previous 4 weeks. The middle figure in each panel shows the reproduction number used to make forecasts over each 4 week forecast horizon. The bottom figure shows the effective reproduction number estimated retrospectively using the full dataset up to the length of one calibration window before the end. In each case, the solid black line is the median estimate and the shaded region represents the 95% Credible Interval. The red horizontal dashed line indicates the $R_t = 1$ threshold. Results are shown for the three mainly affected countries although the analysis was done jointly using data for all countries in Africa.

2.2.2 Forecast horizon of 6 weeks

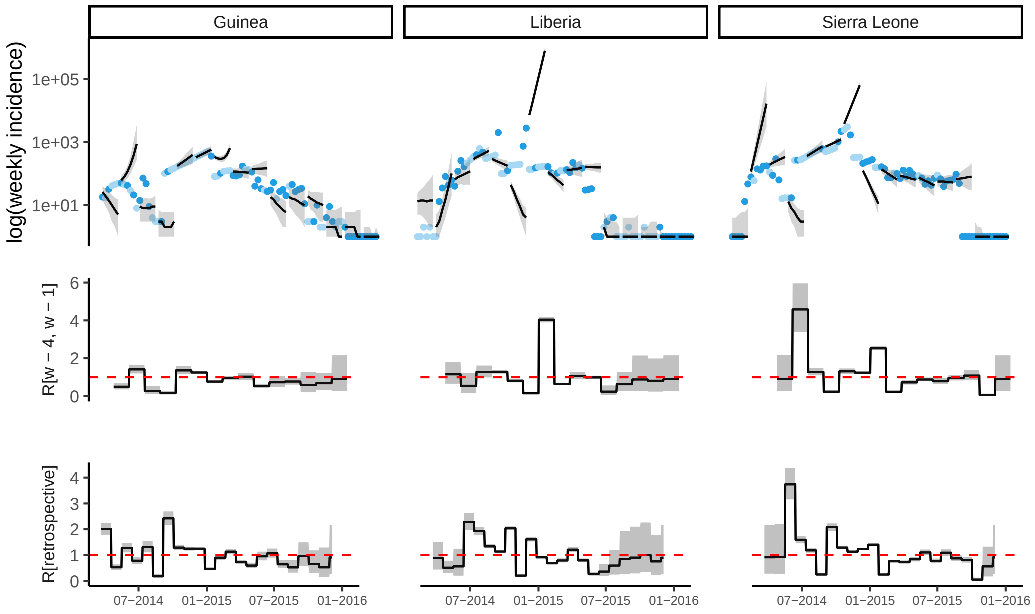

Supplementary Figure 9: Observed and predicted incidence, and reproduction number estimates from ProMED data. The top panel shows the weekly incidence derived from ProMED data and the 6 weeks incidence forecast on log scale. The solid dots represent the observed weekly incidence where the light blue dots show weeks for which all data points were obtained using interpolation. The projections are made over 6 week windows, based on the reproduction number estimated in the previous 4 weeks. The middle figure in each panel shows the reproduction number used to make forecasts over each 6 week forecast horizon. The bottom figure shows the effective reproduction number estimated retrospectively using the full dataset up to the length of one calibration window before the end. In each case, the solid black line is the median estimate and the shaded region represents the 95% Credible Interval. The red horizontal dashed line indicates the $R_t = 1$ threshold. Results are shown for the three mainly affected countries although the analysis was done jointly using data for all countries in Africa.

2.2.3 Forecast horizon of 8 weeks

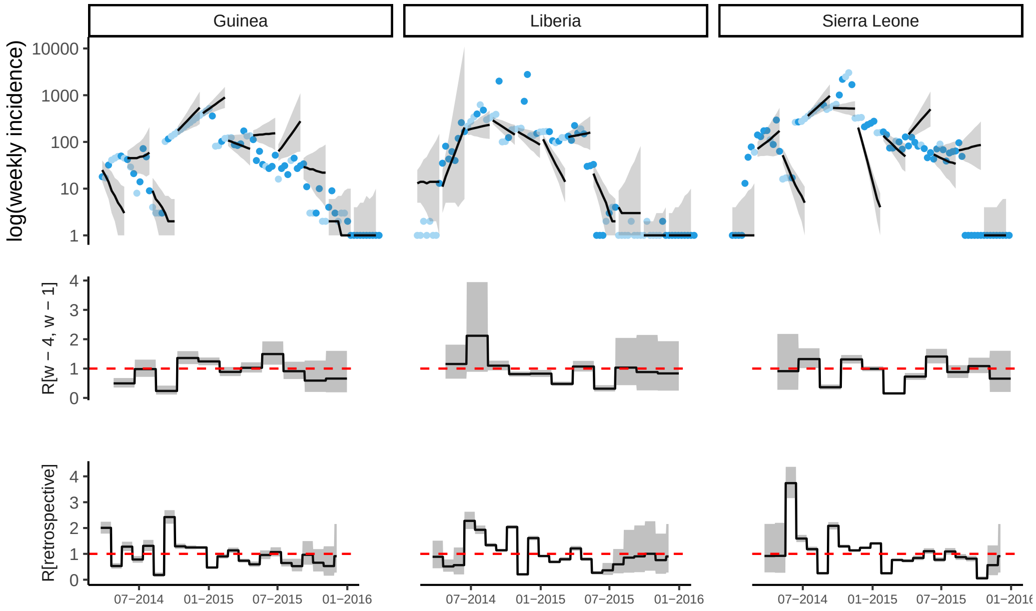

Supplementary Figure 10: Observed and predicted incidence, and reproduction number estimates from ProMED data. The top panel shows the weekly incidence derived from ProMED data and the 8 weeks incidence forecast on log scale. The solid dots represent the observed weekly incidence where the light blue dots show weeks for which all data points were obtained using interpolation. The projections are made over 8 week windows, based on the reproduction number estimated in the previous 4 weeks. The middle figure in each panel shows the reproduction number used to make forecasts over each 8 week forecast horizon. The bottom figure shows the effective reproduction number estimated retrospectively using the full dataset up to the length of one calibration window before the end. In each case, the solid black line is the median estimate and the shaded region represents the 95% Credible Interval. The red horizontal dashed line indicates the $R_t = 1$ threshold. Results are shown for the three mainly affected countries although the analysis was done jointly using data for all countries in Africa.

2.3 Calibration window of 6 weeks

2.3.1 Forecast horizon of 4 weeks

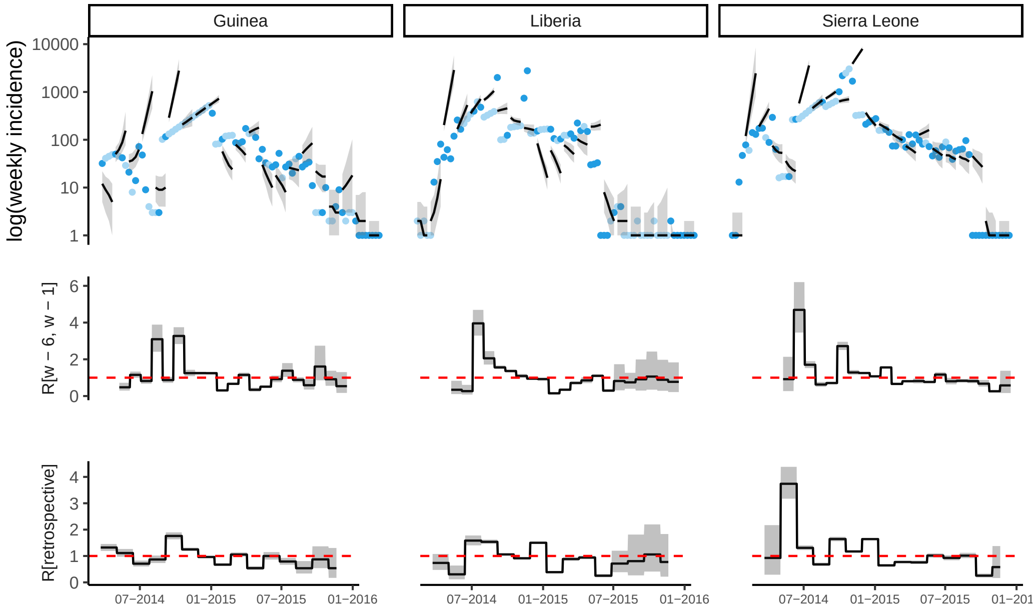

Supplementary Figure 11: Observed and predicted incidence, and reproduction number estimates from ProMED data. The top panel shows the weekly incidence derived from ProMED data and the 4 weeks incidence forecast on log scale. The solid dots represent the observed weekly incidence where the light blue dots show weeks for which all data points were obtained using interpolation. The projections are made over 4 week windows, based on the reproduction number estimated in the previous 6 weeks. The middle figure in each panel shows the reproduction number used to make forecasts over each 4 week forecast horizon. The bottom figure shows the effective reproduction number estimated retrospectively using the full dataset up to the length of one calibration window before the end. In each case, the solid black line is the median estimate and the shaded region represents the 95% Credible Interval. The red horizontal dashed line indicates the $R_t = 1$ threshold. Results are shown for the three mainly affected countries although the analysis was done jointly using data for all countries in Africa.

2.3.2 Forecast horizon of 6 weeks

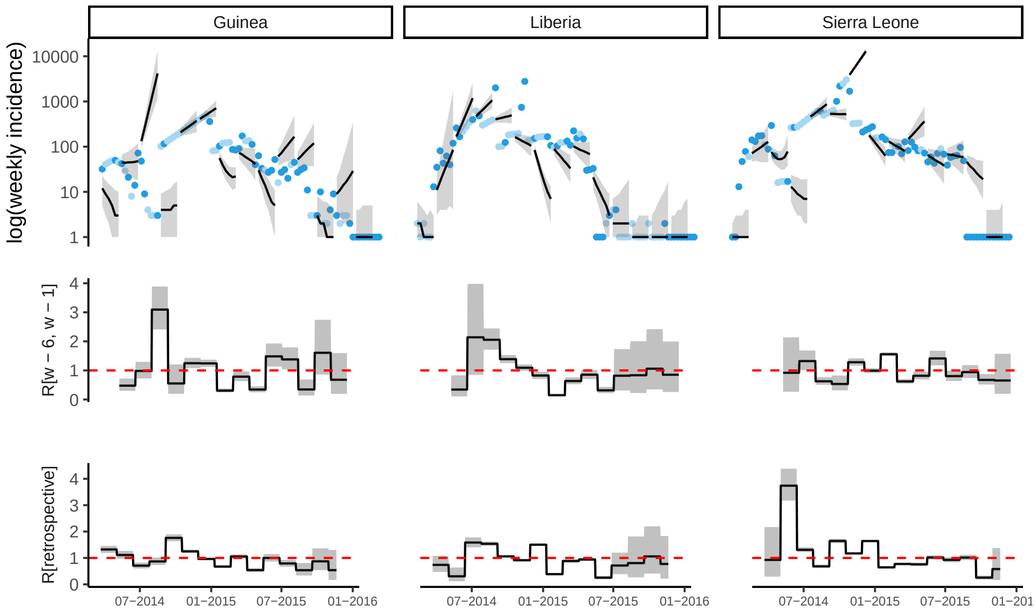

Supplementary Figure 12: Observed and predicted incidence, and reproduction number estimates from ProMED data. The top panel shows the weekly incidence derived from ProMED data and the 6 weeks incidence forecast on log scale. The solid dots represent the observed weekly incidence where the light blue dots show weeks for which all data points were obtained using interpolation. The projections are made over 6 week windows, based on the reproduction number estimated in the previous 6 weeks. The middle figure in each panel shows the reproduction number used to make forecasts over each 6 week forecast horizon. The bottom figure shows the effective reproduction number estimated retrospectively using the full dataset up to the length of one calibration window before the end. In each case, the solid black line is the median estimate and the shaded region represents the 95% Credible Interval. The red horizontal dashed line indicates the $R_t = 1$ threshold. Results are shown for the three mainly affected countries although the analysis was done jointly using data for all countries in Africa.

2.3.3 Forecast horizon of 8 weeks

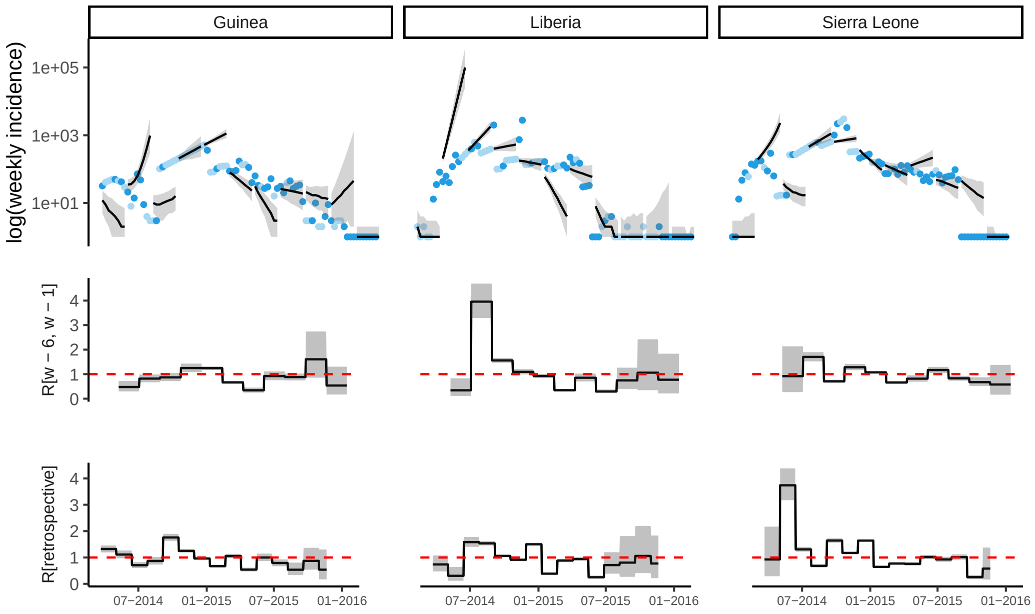

Supplementary Figure 13: Observed and predicted incidence, and reproduction number estimates from ProMED data. The top panel shows the weekly incidence derived from ProMED data and the 8 weeks incidence forecast on log scale. The solid dots represent the observed weekly incidence where the light blue dots show weeks for which all data points were obtained using interpolation. The projections are made over 8 week windows, based on the reproduction number estimated in the previous 6 weeks. The middle figure in each panel shows the reproduction number used to make forecasts over each 8 week forecast horizon. The bottom figure shows the effective reproduction number estimated retrospectively using the full dataset up to the length of one calibration window before the end. In each case, the solid black line is the median estimate and the shaded region represents the 95% Credible Interval. The red horizontal dashed line indicates the $R_t = 1$ threshold. Results are shown for the three mainly affected countries although the analysis was done jointly using data for all countries in Africa.

3 Forecasts using HealthMap data

This section presents the forecasts over 4, 6 and 8 weeks produced using HealthMap data and calibration window of 2, 4 and 6 weeks.

3.1 Calibration window of 2 weeks

3.1.1 Forecast horizon of 4 weeks

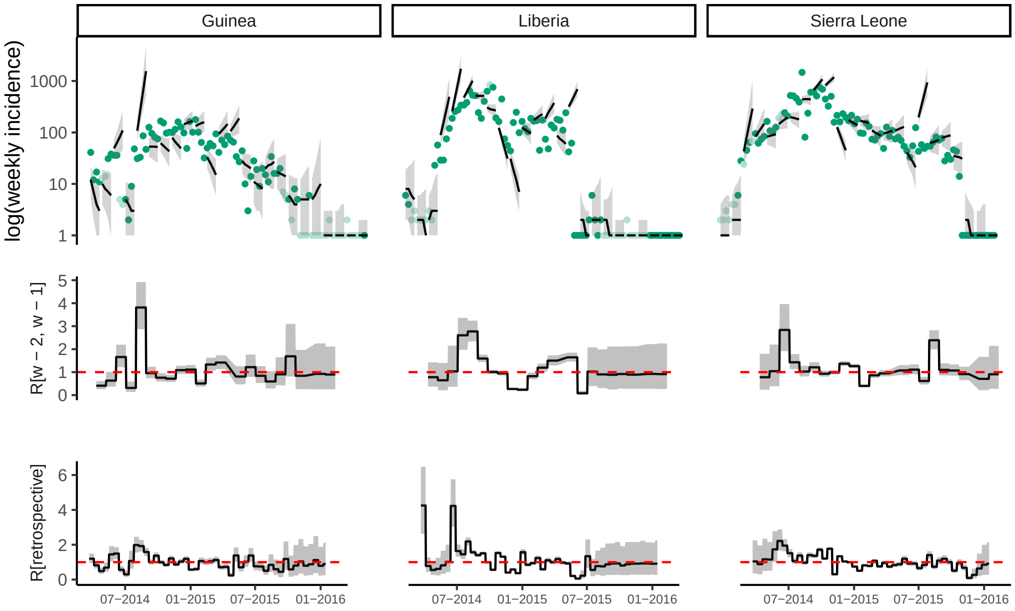

Supplementary Figure 14: Observed and predicted incidence, and reproduction number estimates from HealthMap data. The top panel shows the weekly incidence derived from HealthMap data and the 4 weeks incidence forecast on log scale. The solid dots represent the observed weekly incidence where the light green dots show weeks for which all data points were obtained using interpolation. The projections are made over 4 week windows, based on the reproduction number estimated in the previous 2 weeks. The middle figure in each panel shows the reproduction number used to make forecasts over each 4 week forecast horizon. The bottom figure shows the effective reproduction number estimated retrospectively using the full dataset up to the length of one calibration window before the end. In each case, the solid black line is the median estimate and the shaded region represents the 95% Credible Interval. The red horizontal dashed line indicates the $R_t = 1$ threshold. Results are shown for the three mainly affected countries although the analysis was done jointly using data for all countries in Africa.

3.1.2 Forecast horizon of 6 weeks

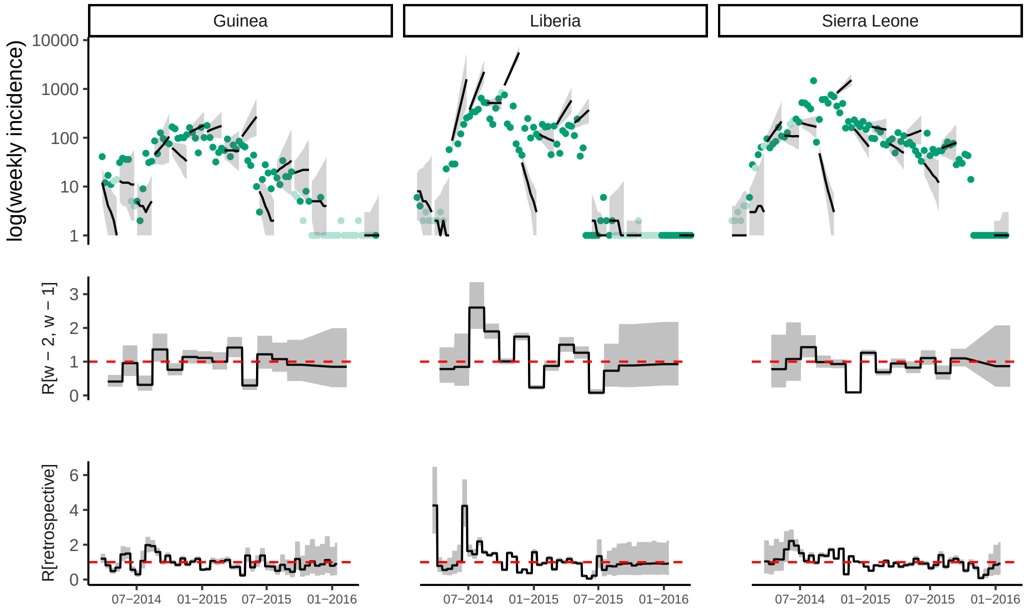

Supplementary Figure 15: Observed and predicted incidence, and reproduction number estimates from HealthMap data. The top panel shows the weekly incidence derived from HealthMap data and the 6 weeks incidence forecast on log scale. The solid dots represent the observed weekly incidence where the light green dots show weeks for which all data points were obtained using interpolation. The projections are made over 6 week windows, based on the reproduction number estimated in the previous 2 weeks. The middle figure in each panel shows the reproduction number used to make forecasts over each 6 week forecast horizon. The bottom figure shows the effective reproduction number estimated retrospectively using the full dataset up to the length of one calibration window before the end. In each case, the solid black line is the median estimate and the shaded region represents the 95% Credible Interval. The red horizontal dashed line indicates the $R_t = 1$ threshold. Results are shown for the three mainly affected countries although the analysis was done jointly using data for all countries in Africa.

3.1.3 Forecast horizon of 8 weeks

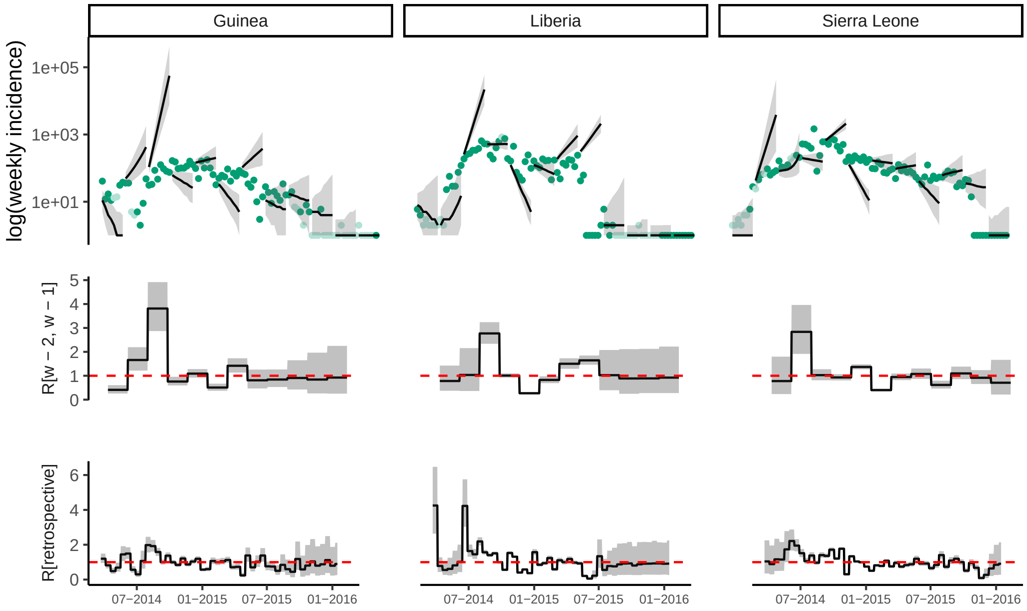

Supplementary Figure 16: Observed and predicted incidence, and reproduction number estimates from HealthMap data. The top panel shows the weekly incidence derived from HealthMap data and the 8 weeks incidence forecast on log scale. The solid dots represent the observed weekly incidence where the light green dots show weeks for which all data points were obtained using interpolation. The projections are made over 8 week windows, based on the reproduction number estimated in the previous 2 weeks. The middle figure in each panel shows the reproduction number used to make forecasts over each 8 week forecast horizon. The bottom figure shows the effective reproduction number estimated retrospectively using the full dataset up to the length of one calibration window before the end. In each case, the solid black line is the median estimate and the shaded region represents the 95% Credible Interval. The red horizontal dashed line indicates the $R_t = 1$ threshold. Results are shown for the three mainly affected countries although the analysis was done jointly using data for all countries in Africa.

3.2 Calibration window of 4 weeks

3.2.1 Forecast horizon of 4 weeks

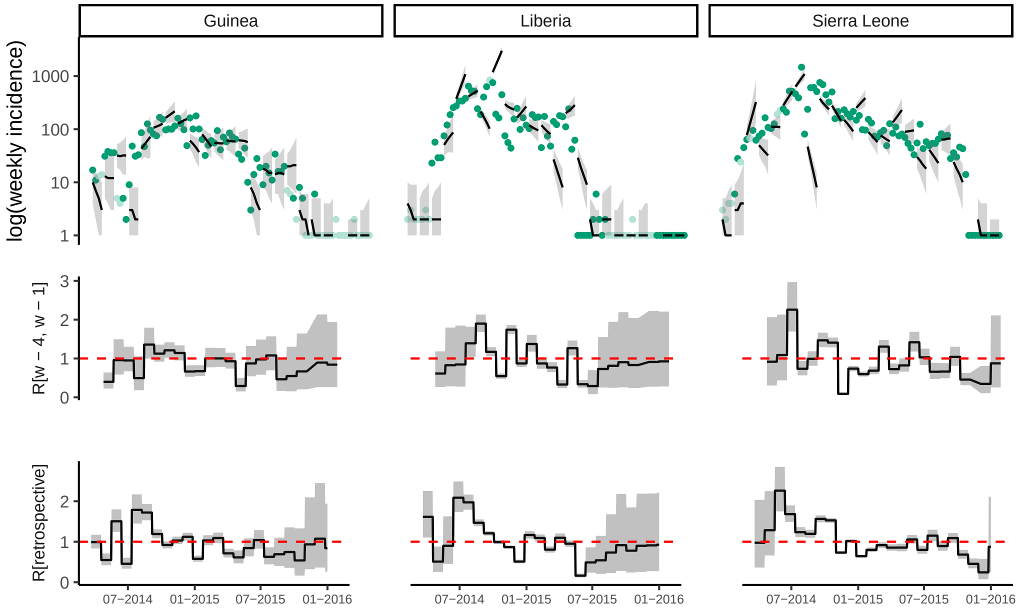

Supplementary Figure 17: Observed and predicted incidence, and reproduction number estimates from HealthMap data. The top panel shows the weekly incidence derived from HealthMap data and the 4 weeks incidence forecast on log scale. The solid dots represent the observed weekly incidence where the light green dots show weeks for which all data points were obtained using interpolation. The projections are made over 4 week windows, based on the reproduction number estimated in the previous 4 weeks. The middle figure in each panel shows the reproduction number used to make forecasts over each 4 week forecast horizon. The bottom figure shows the effective reproduction number estimated retrospectively using the full dataset up to the length of one calibration window before the end. In each case, the solid black line is the median estimate and the shaded region represents the 95% Credible Interval. The red horizontal dashed line indicates the $R_t = 1$ threshold. Results are shown for the three mainly affected countries although the analysis was done jointly using data for all countries in Africa.

3.2.2 Forecast horizon of 6 weeks

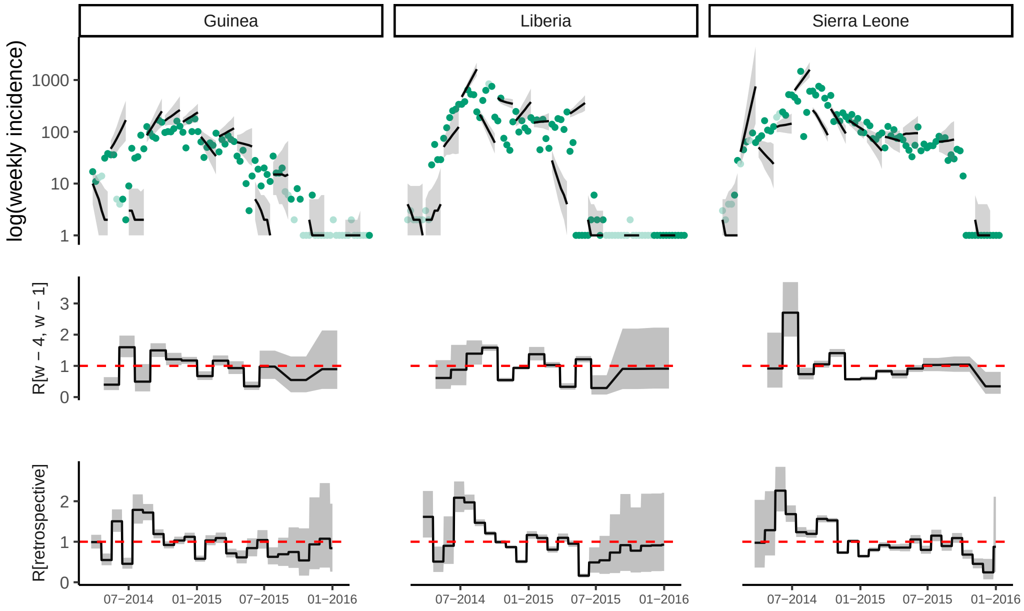

Supplementary Figure 18: Observed and predicted incidence, and reproduction number estimates from HealthMap data. The top panel shows the weekly incidence derived from HealthMap data and the 6 weeks incidence forecast on log scale. The solid dots represent the observed weekly incidence where the light green dots show weeks for which all data points were obtained using interpolation. The projections are made over 6 week windows, based on the reproduction number estimated in the previous 4 weeks. The middle figure in each panel shows the reproduction number used to make forecasts over each 6 week forecast horizon. The bottom figure shows the effective reproduction number estimated retrospectively using the full dataset up to the length of one calibration window before the end. In each case, the solid black line is the median estimate and the shaded region represents the 95% Credible Interval. The red horizontal dashed line indicates the $R_t = 1$ threshold. Results are shown for the three mainly affected countries although the analysis was done jointly using data for all countries in Africa.

3.2.3 Forecast horizon of 8 weeks

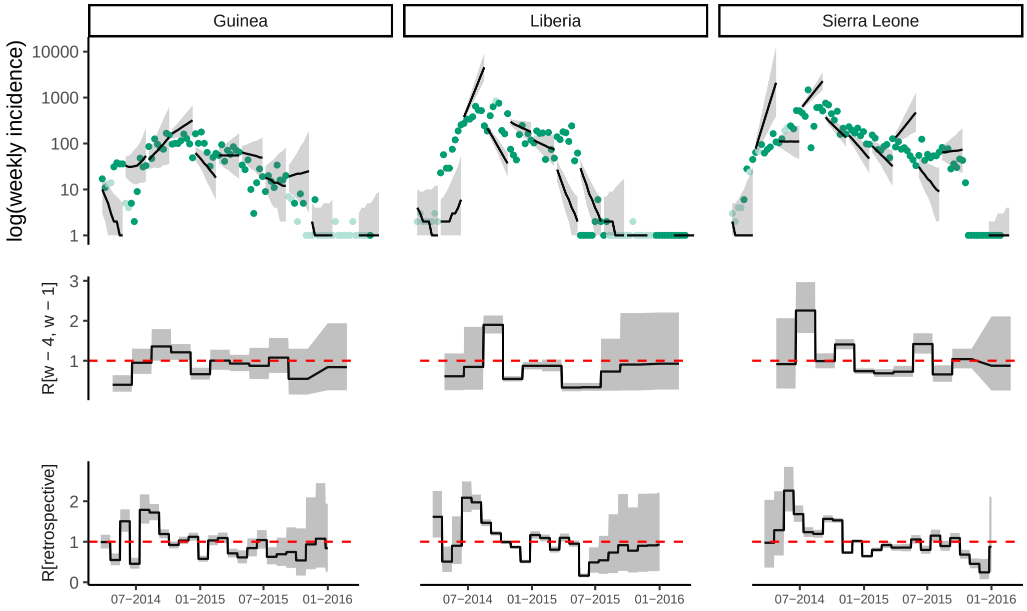

Supplementary Figure 19: Observed and predicted incidence, and reproduction number estimates from HealthMap data. The top panel shows the weekly incidence derived from HealthMap data and the 8 weeks incidence forecast on log scale. The solid dots represent the observed weekly incidence where the light green dots show weeks for which all data points were obtained using interpolation. The projections are made over 8 week windows, based on the reproduction number estimated in the previous 4 weeks. The middle figure in each panel shows the reproduction number used to make forecasts over each 8 week forecast horizon. The bottom figure shows the effective reproduction number estimated retrospectively using the full dataset up to the length of one calibration window before the end. In each case, the solid black line is the median estimate and the shaded region represents the 95% Credible Interval. The red horizontal dashed line indicates the $R_t = 1$ threshold. Results are shown for the three mainly affected countries although the analysis was done jointly using data for all countries in Africa.

3.3 Calibration window of 6 weeks

3.3.1 Forecast horizon of 4 weeks

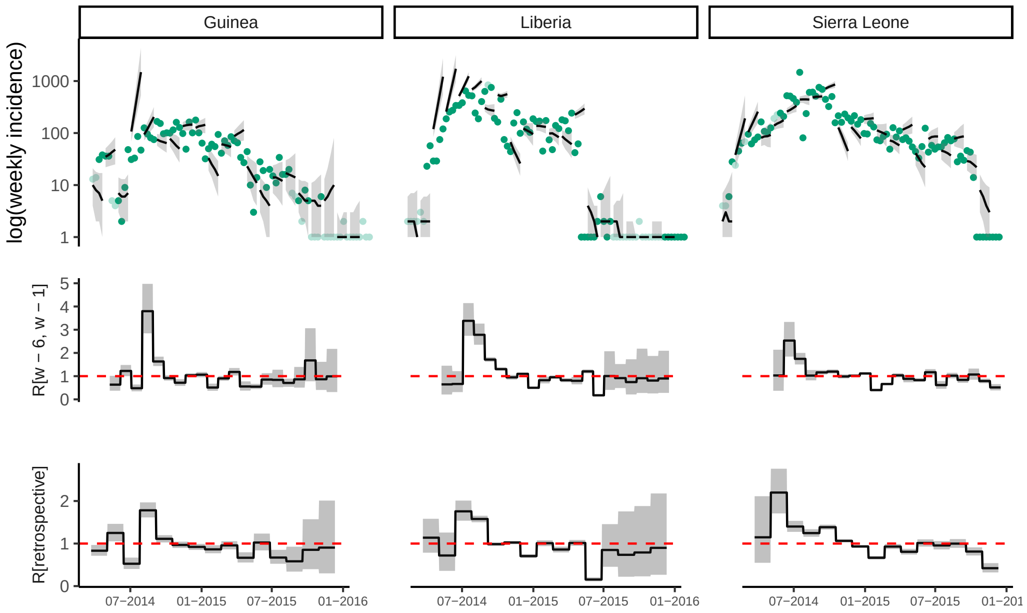

Supplementary Figure 20: Observed and predicted incidence, and reproduction number estimates from HealthMap data. The top panel shows the weekly incidence derived from HealthMap data and the 4 weeks incidence forecast on log scale. The solid dots represent the observed weekly incidence where the light green dots show weeks for which all data points were obtained using interpolation. The projections are made over 4 week windows, based on the reproduction number estimated in the previous 6 weeks. The middle figure in each panel shows the reproduction number used to make forecasts over each 4 week forecast horizon. The bottom figure shows the effective reproduction number estimated retrospectively using the full dataset up to the length of one calibration window before the end. In each case, the solid black line is the median estimate and the shaded region represents the 95% Credible Interval. The red horizontal dashed line indicates the $R_t = 1$ threshold. Results are shown for the three mainly affected countries although the analysis was done jointly using data for all countries in Africa.

3.3.2 Forecast horizon of 6 weeks

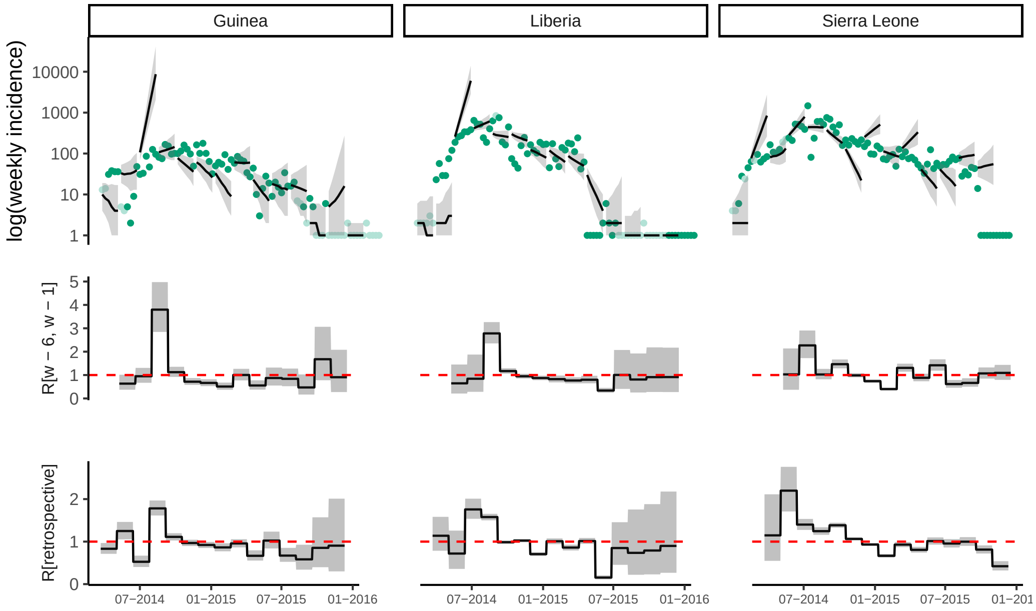

Supplementary Figure 21: Observed and predicted incidence, and reproduction number estimates from HealthMap data. The top panel shows the weekly incidence derived from HealthMap data and the 6 weeks incidence forecast on log scale. The solid dots represent the observed weekly incidence where the light green dots show weeks for which all data points were obtained using interpolation. The projections are made over 6 week windows, based on the reproduction number estimated in the previous 6 weeks. The middle figure in each panel shows the reproduction number used to make forecasts over each 6 week forecast horizon. The bottom figure shows the effective reproduction number estimated retrospectively using the full dataset up to the length of one calibration window before the end. In each case, the solid black line is the median estimate and the shaded region represents the 95% Credible Interval. The red horizontal dashed line indicates the $R_t = 1$ threshold. Results are shown for the three mainly affected countries although the analysis was done jointly using data for all countries in Africa.

3.3.3 Forecast horizon of 8 weeks

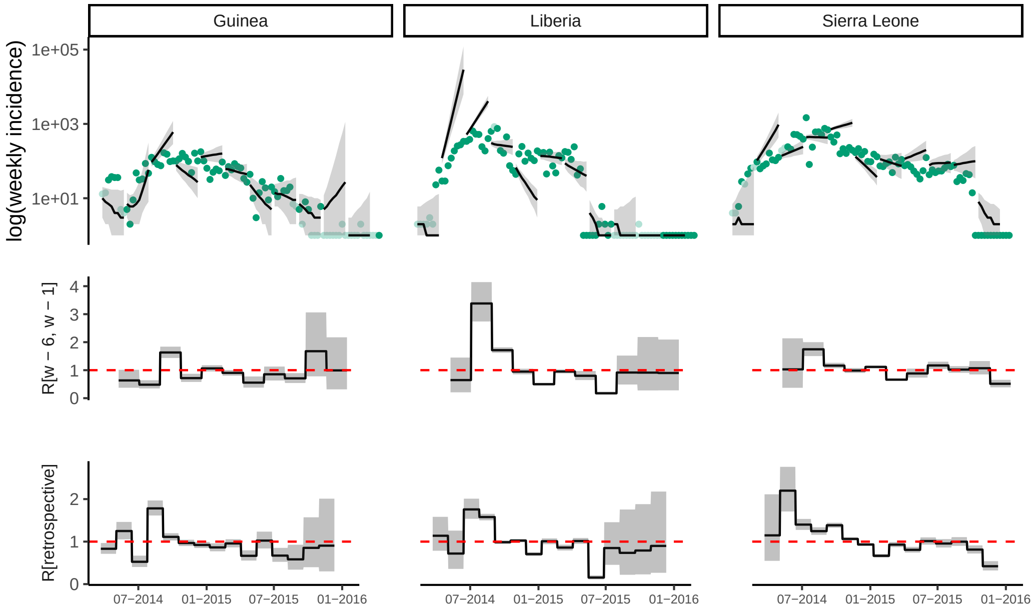

Supplementary Figure 22: Observed and predicted incidence, and reproduction number estimates from HealthMap data. The top panel shows the weekly incidence derived from HealthMap data and the 8 weeks incidence forecast on log scale. The solid dots represent the observed weekly incidence where the light green dots show weeks for which all data points were obtained using interpolation. The projections are made over 8 week windows, based on the reproduction number estimated in the previous 6 weeks. The middle figure in each panel shows the reproduction number used to make forecasts over each 8 week forecast horizon. The bottom figure shows the effective reproduction number estimated retrospectively using the full dataset up to the length of one calibration window before the end. In each case, the solid black line is the median estimate and the shaded region represents the 95% Credible Interval. The red horizontal dashed line indicates the $R_t = 1$ threshold. Results are shown for the three mainly affected countries although the analysis was done jointly using data for all countries in Africa.

4 Forecasts using WHO data

This section presents the model forecasts over 4 6 and 6 weeks horizon produced using WHO data and calibration window of 2, 4 or 6 weeks.

4.1 Calibration window of 2 weeks

4.1.1 Forecast horizon of 4 weeks

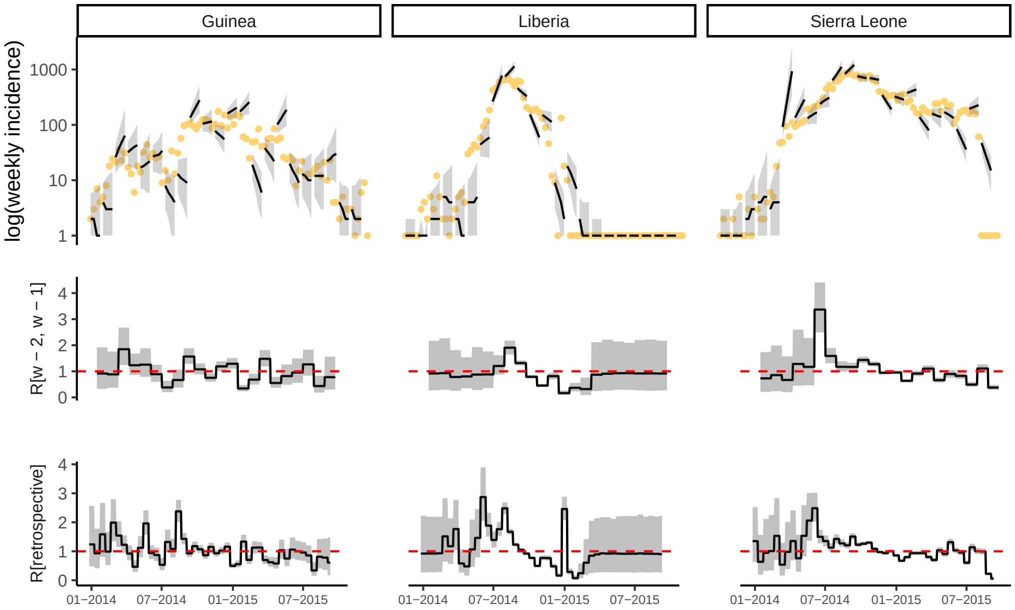

Supplementary Figure 23: Observed and predicted incidence, and reproduction number estimates from WHO data. The top panel shows the weekly incidence derived from WHO data and the 4 weeks incidence forecast on log scale. The solid dots represent the observed weekly incidence. The projections are made over 4 week windows, based on the reproduction number estimated in the previous 2 weeks. The middle figure in each panel shows the reproduction number used to make forecasts over each 4 week forecast horizon. The bottom figure shows the effective reproduction number estimated retrospectively using the full dataset up to the length of one calibration window before the end. In each case, the solid black line is the median estimate and the shaded region represents the 95% Credible Interval. The red horizontal dashed line indicates the $R_t = 1$ threshold. Results are shown for the three mainly affected countries although the analysis was done jointly using data for all countries in Africa.

4.1.2 Forecast horizon of 6 weeks

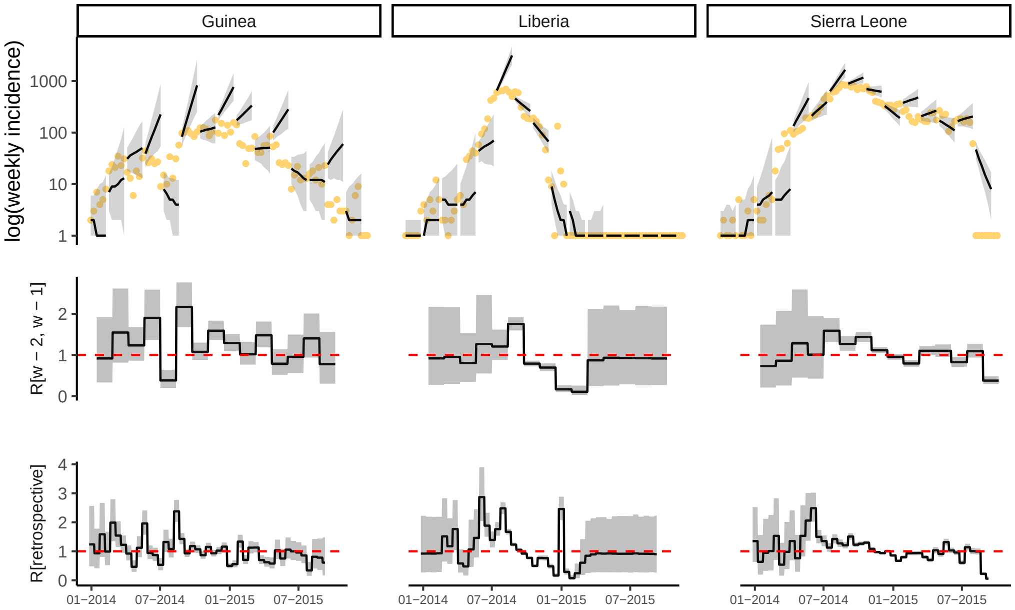

Supplementary Figure 24: Observed and predicted incidence, and reproduction number estimates from WHO data. The top panel shows the weekly incidence derived from WHO data and the 6 weeks incidence forecast on log scale. The solid dots represent the observed weekly incidence. The projections are made over 6 week windows, based on the reproduction number estimated in the previous 2 weeks. The middle figure in each panel shows the reproduction number used to make forecasts over each 6 week forecast horizon. The bottom figure shows the effective reproduction number estimated retrospectively using the full dataset up to the length of one calibration window before the end. In each case, the solid black line is the median estimate and the shaded region represents the 95% Credible Interval. The red horizontal dashed line indicates the $R_t = 1$ threshold. Results are shown for the three mainly affected countries although the analysis was done jointly using data for all countries in Africa.

4.1.3 Forecast horizon of 8 weeks

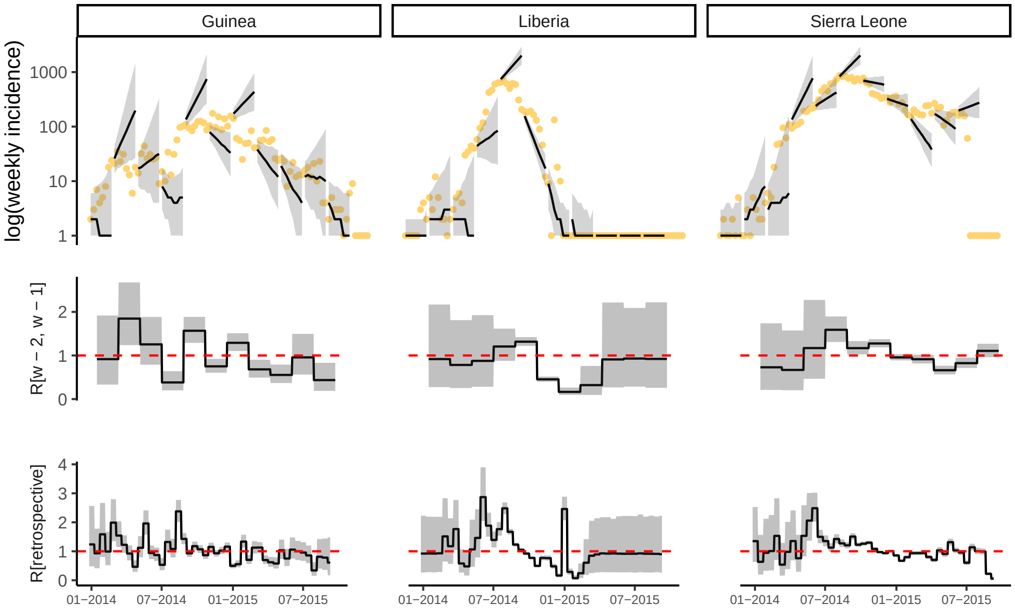

Supplementary Figure 25: Observed and predicted incidence, and reproduction number estimates from WHO data. The top panel shows the weekly incidence derived from WHO data and the 8 weeks incidence forecast on log scale. The solid dots represent the observed weekly incidence. The projections are made over 8 week windows, based on the reproduction number estimated in the previous 2 weeks. The middle figure in each panel shows the reproduction number used to make forecasts over each 8 week forecast horizon. The bottom figure shows the effective reproduction number estimated retrospectively using the full dataset up to the length of one calibration window before the end. In each case, the solid black line is the median estimate and the shaded region represents the 95% Credible Interval. The red horizontal dashed line indicates the $R_t = 1$ threshold. Results are shown for the three mainly affected countries although the analysis was done jointly using data for all countries in Africa.

4.2 Calibration window of 4 weeks

4.2.1 Forecast horizon of 4 weeks

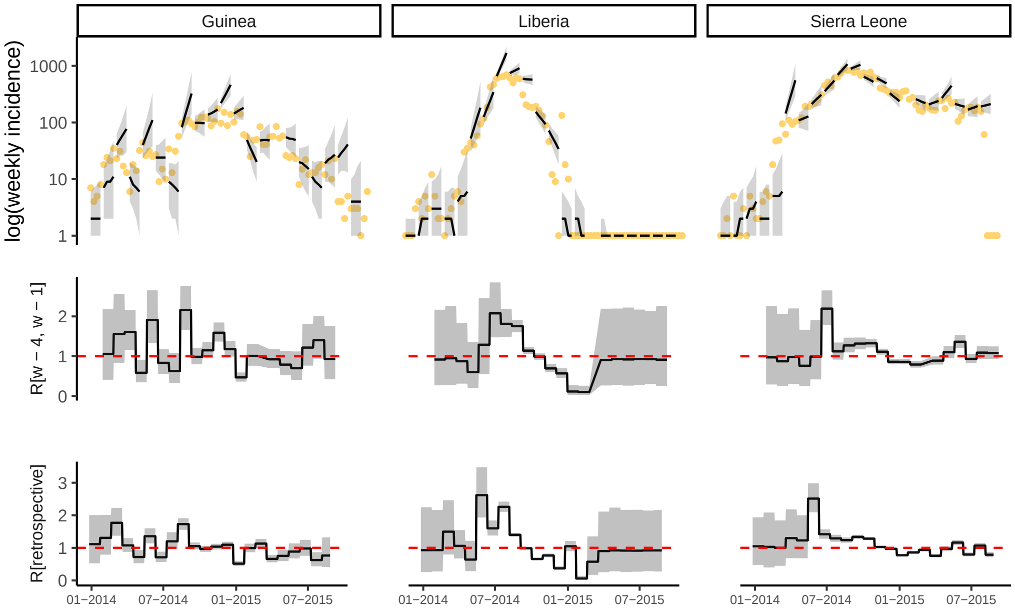

Supplementary Figure 26: Observed and predicted incidence, and reproduction number estimates from WHO data. The top panel shows the weekly incidence derived from WHO data and the 4 weeks incidence forecast on log scale. The solid dots represent the observed weekly incidence. The projections are made over 4 week windows, based on the reproduction number estimated in the previous 4 weeks. The middle figure in each panel shows the reproduction number used to make forecasts over each 4 week forecast horizon. The bottom figure shows the effective reproduction number estimated retrospectively using the full dataset up to the length of one calibration window before the end. In each case, the solid black line is the median estimate and the shaded region represents the 95% Credible Interval. The red horizontal dashed line indicates the $R_t = 1$ threshold. Results are shown for the three mainly affected countries although the analysis was done jointly using data for all countries in Africa.

4.2.2 Forecast horizon of 6 weeks

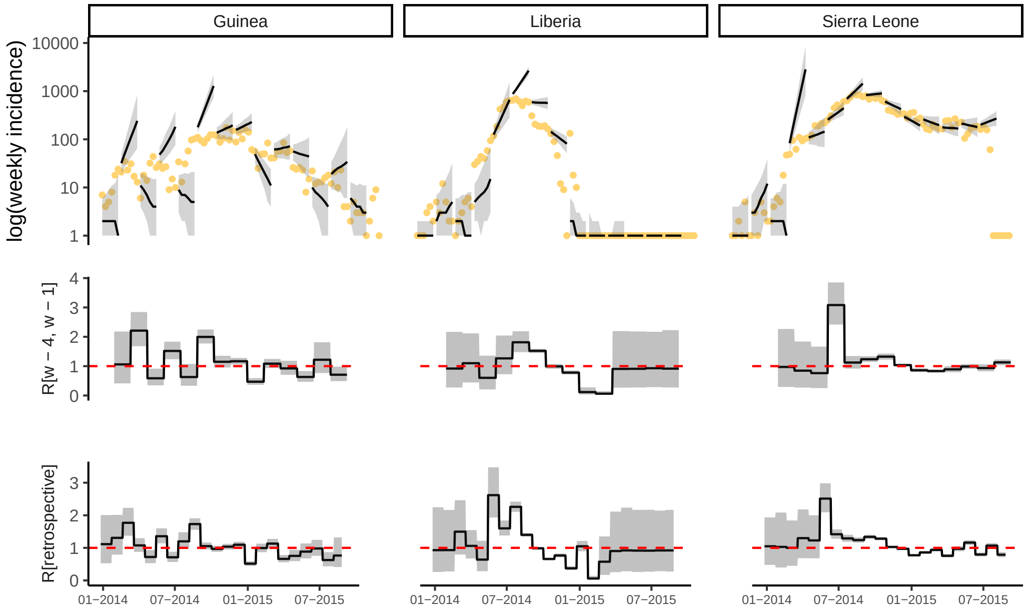

Supplementary Figure 27: Observed and predicted incidence, and reproduction number estimates from WHO data. The top panel shows the weekly incidence derived from WHO data and the 6 weeks incidence forecast on log scale. The solid dots represent the observed weekly incidence. The projections are made over 6 week windows, based on the reproduction number estimated in the previous 4 weeks. The middle figure in each panel shows the reproduction number used to make forecasts over each 6 week forecast horizon. The bottom figure shows the effective reproduction number estimated retrospectively using the full dataset up to the length of one calibration window before the end. In each case, the solid black line is the median estimate and the shaded region represents the 95% Credible Interval. The red horizontal dashed line indicates the $R_t = 1$ threshold. Results are shown for the three mainly affected countries although the analysis was done jointly using data for all countries in Africa.

4.2.3 Forecast horizon of 8 weeks

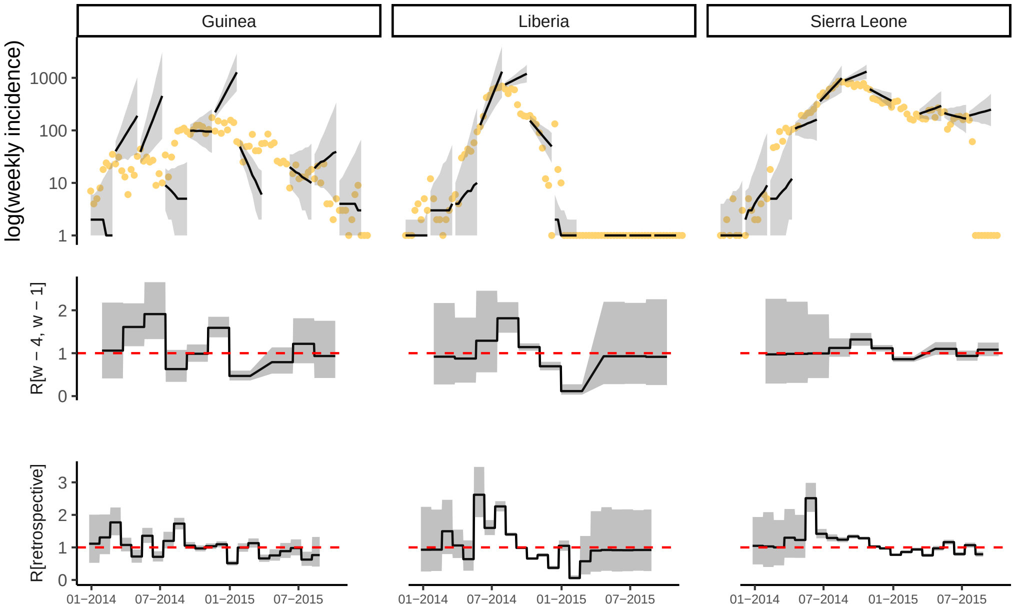

Supplementary Figure 28: Observed and predicted incidence, and reproduction number estimates from WHO data. The top panel shows the weekly incidence derived from WHO data and the 8 weeks incidence forecast on log scale. The solid dots represent the observed weekly incidence. The projections are made over 8 week windows, based on the reproduction number estimated in the previous 4 weeks. The middle figure in each panel shows the reproduction number used to make forecasts over each 8 week forecast horizon. The bottom figure shows the effective reproduction number estimated retrospectively using the full dataset up to the length of one calibration window before the end. In each case, the solid black line is the median estimate and the shaded region represents the 95% Credible Interval. The red horizontal dashed line indicates the $R_t = 1$ threshold. Results are shown for the three mainly affected countries although the analysis was done jointly using data for all countries in Africa.

4.3 Calibration window of 6 weeks

4.3.1 Forecast horizon of 4 weeks

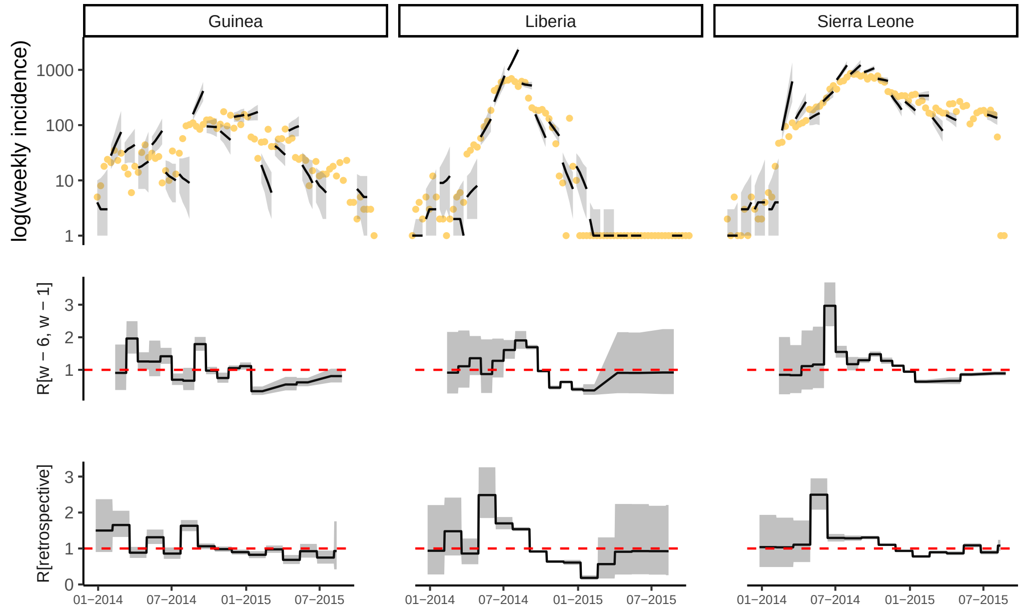

Supplementary Figure 29: Observed and predicted incidence, and reproduction number estimates from WHO data. The top panel shows the weekly incidence derived from WHO data and the 4 weeks incidence forecast on log scale. The solid dots represent the observed weekly incidence. The projections are made over 4 week windows, based on the reproduction number estimated in the previous 6 weeks. The middle figure in each panel shows the reproduction number used to make forecasts over each 4 week forecast horizon. The bottom figure shows the effective reproduction number estimated retrospectively using the full dataset up to the length of one calibration window before the end. In each case, the solid black line is the median estimate and the shaded region represents the 95% Credible Interval. The red horizontal dashed line indicates the $R_t = 1$ threshold. Results are shown for the three mainly affected countries although the analysis was done jointly using data for all countries in Africa.

4.3.2 Forecast horizon of 6 weeks

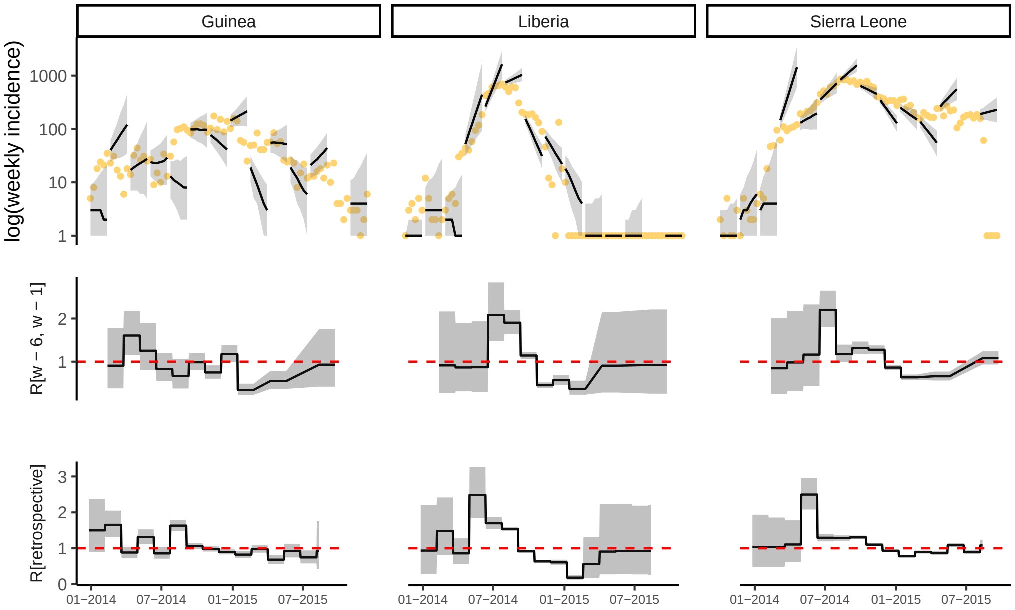

Supplementary Figure 30: Observed and predicted incidence, and reproduction number estimates from WHO data. The top panel shows the weekly incidence derived from WHO data and the 6 weeks incidence forecast on log scale. The solid dots represent the observed weekly incidence. The projections are made over 6 week windows, based on the reproduction number estimated in the previous 6 weeks. The middle figure in each panel shows the reproduction number used to make forecasts over each 6 week forecast horizon. The bottom figure shows the effective reproduction number estimated retrospectively using the full dataset up to the length of one calibration window before the end. In each case, the solid black line is the median estimate and the shaded region represents the 95% Credible Interval. The red horizontal dashed line indicates the $R_t = 1$ threshold. Results are shown for the three mainly affected countries although the analysis was done jointly using data for all countries in Africa.

4.3.3 Forecast horizon of 8 weeks

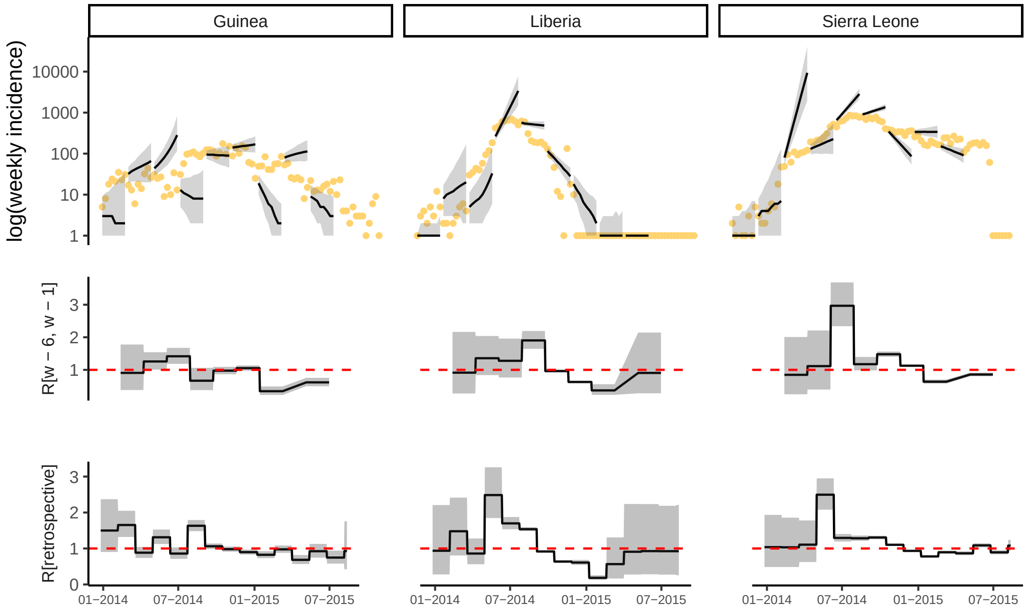

Supplementary Figure 31: Observed and predicted incidence, and reproduction number estimates from WHO data. The top panel shows the weekly incidence derived from WHO data and the 8 weeks incidence forecast on log scale. The solid dots represent the observed weekly incidence. The projections are made over 8 week windows, based on the reproduction number estimated in the previous 6 weeks. The middle figure in each panel shows the reproduction number used to make forecasts over each 8 week forecast horizon. The bottom figure shows the effective reproduction number estimated retrospectively using the full dataset up to the length of one calibration window before the end. In each case, the solid black line is the median estimate and the shaded region represents the 95% Credible Interval. The red horizontal dashed line indicates the $R_t = 1$ threshold. Results are shown for the three mainly affected countries although the analysis was done jointly using data for all countries in Africa.

5 Impact of calibration window on model performance

Since changing the length of the calibration window modified the model complexity with shorter windows introducing more parameters in the model, we assessed the impact of this length on the performance of the model (Supplementary Figure 32).

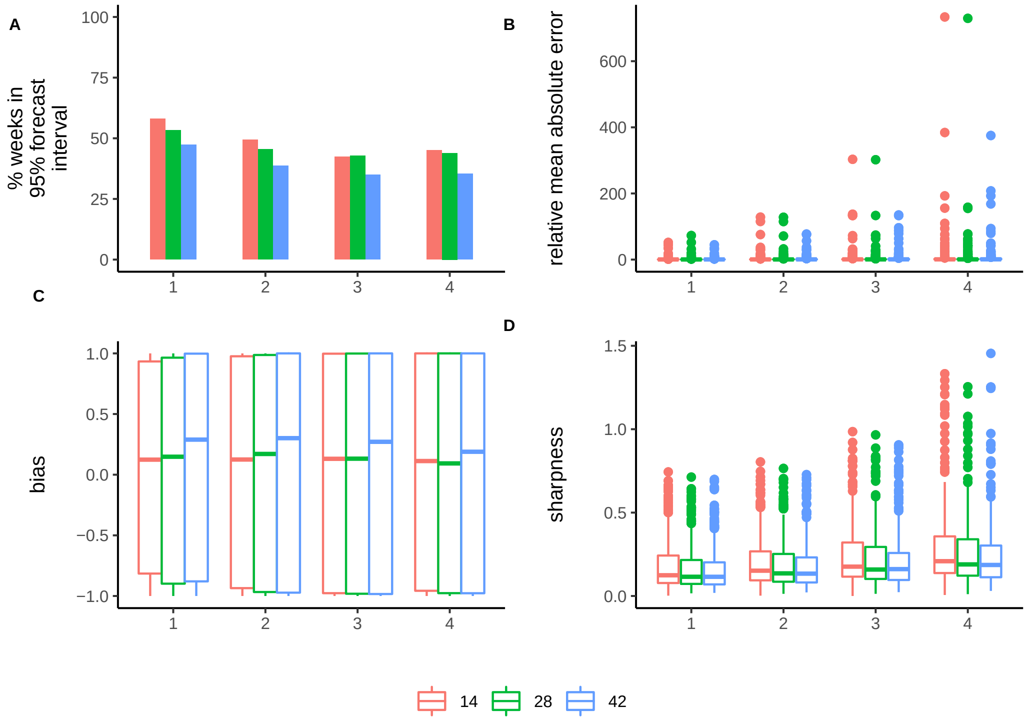

Supplementary Figure 32: Model performance metrics stratified by the time window used for model calibration and week of projection. The performance metrics are (A) the percentage of weeks for which the 95% forecast interval contained the observed incidence, (B) relative mean absolute error, (C) bias, and (D) sharpness. See Methods for details.

6 Impact of datasource on model performance

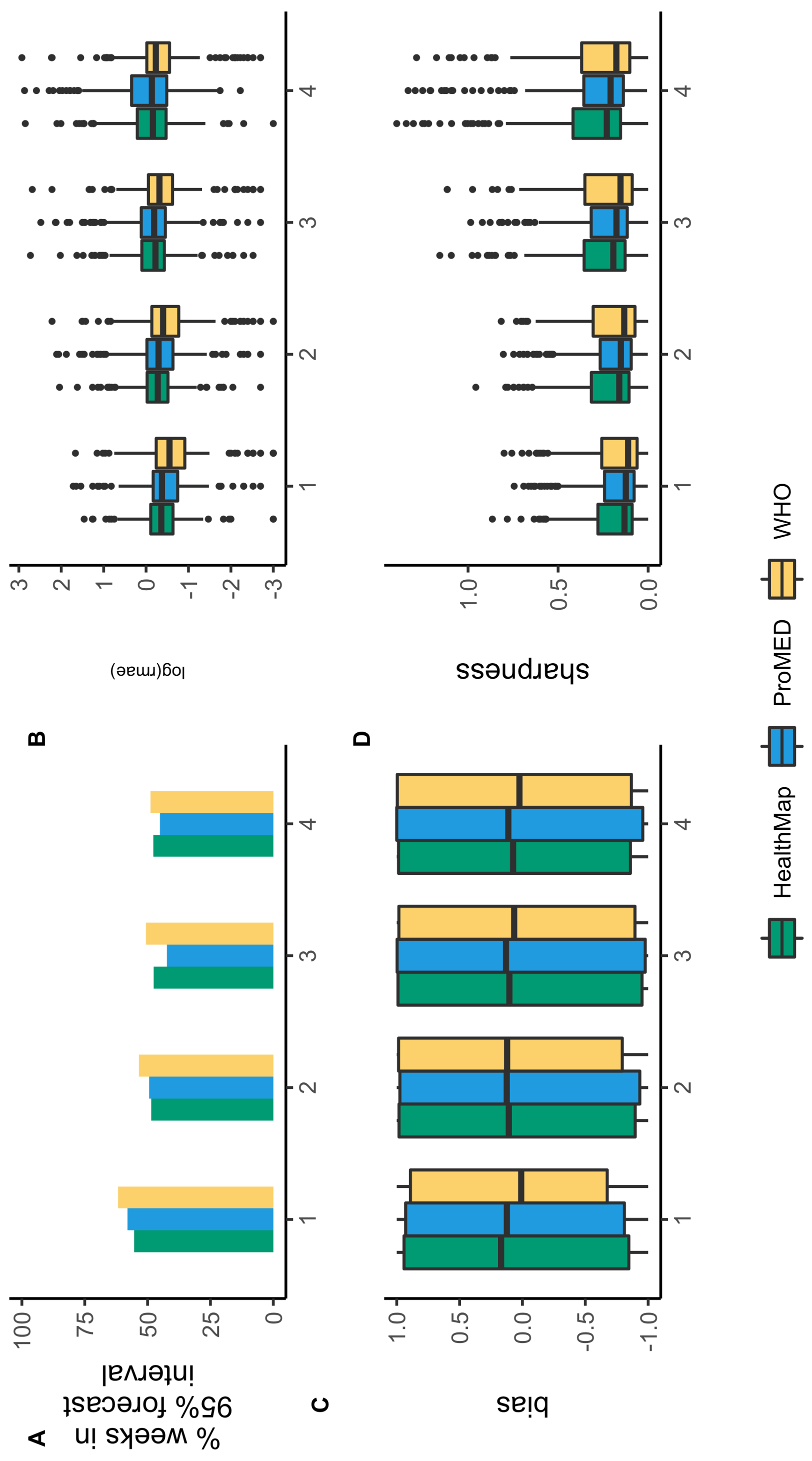

Supplementary Figure 33: Model performance metrics stratified by datasource ProMED (blue), HealthMap (green), and WHO (yellow) and week of projection. The performance metrics are (A) the percentage of weeks for which the 95% forecast interval contained the observed incidence, (B) relative mean absolute error, (C) bias, and (D) sharpness. See Methods for details.

7 Mobility Model Parameters

We estimated the parameters of gravity model - p_{stay} which is the probability of an infectious case staying within a country, and γ which measures the extent to which distance between two locations influences the flow of people between them (Supplementary Figure 34).

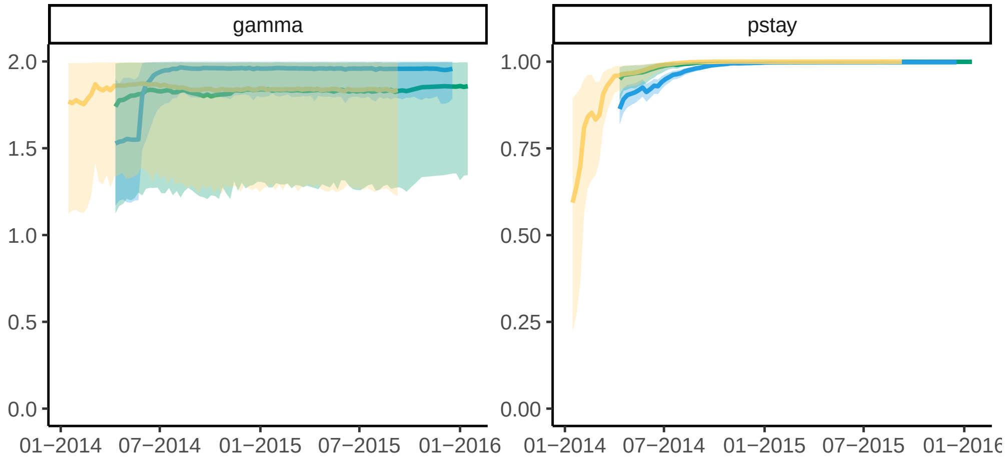

Supplementary Figure 34: Estimates of mobility model parameters during the epidemic. Population movement was modelled using a gravity model where the flow between locations i and j is proportional to the product of their populations and inversely population to the distance between them raised to an exponent γ. The parameter γ thus modulates the influence of distance on the population flow. p_{stay} represents the probability of an individual to stay in a given location during their infectious period. The solid lines represents the median estimates obtained using WHO (yellow), ProMED (blue) and HealthMap (green) data. The shaded regions represent the 95% CrI.

8 Sensitivity Analysis

For the results presented in the main text, we choose an uninformative uniform prior for the parameter γ with an upper bound 2. We also fitted the model with a uniform prior for γ allowing it to vary from 1 to 10. In this section we present the results from the sensitivity analyses. Supplementary

Figure 34 presents the estimates of the parameters over the course of the epidemic using the alternative priors. As the epidemic progressed, the parameter p_{stay} assumed larger values suggesting a decreased probability of travel over time (Supplementary Figures 34, 35). As p_{stay} assumes large values, the estimated flow is more strongly influenced by p_{stay} than by γ. Furthermore, p_{stay} is likely to depend on the spatial scale of the model. Our analyses were carried out at the national scale; we expect that γ will be more sensitive to p_{stay} at a finer spatial resolution. Overall, the flow between locations using the parameters estimated using the two alternative priors did not vary much (Supplementary Figure 36). Supplementary Figures 37 to 45 present the model forecasts using the alternative priors for γ and Supplementary Figure 46 presents a comparison of model performance metrics using the two priors. Although the analysis was carried out for the three data sources (ProMED, HealthMap and WHO), for brevity we present results using ProMED data only.

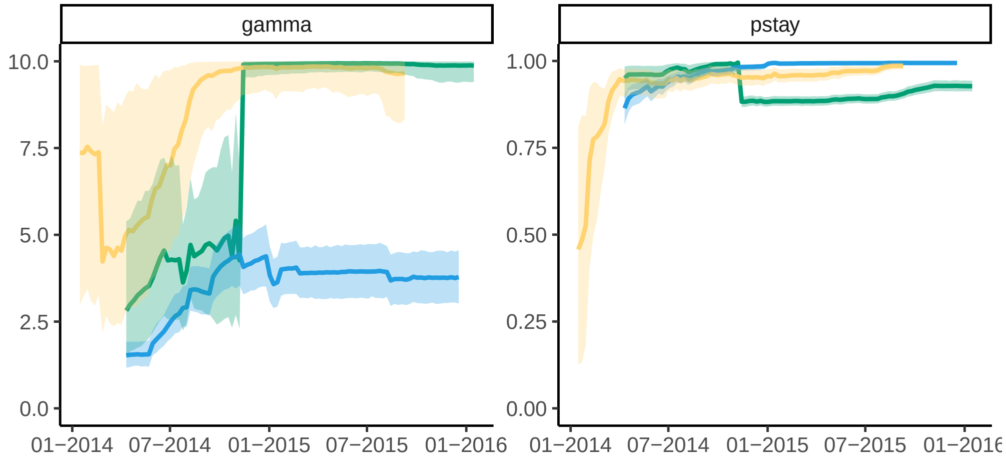

Supplementary Figure 35: Estimates of mobility model parameters during the epidemic. Population movement was modelled using a gravity model where the flow between locations i and j is proportional to the product of their populations and inversely population to the distance between them raised to an exponent γ. The parameter γ thus modulates the influence of distance on the population flow. Here γ is allowed to vary between 1 and 10. p_{stay} represents the probability of an individual to stay in a given location during their infectious period. The solid lines represents the median estimates obtained using ProMED (blue), HealthMap (green) and WHO (yellow) data. The shaded regions represent the 95% CrI.

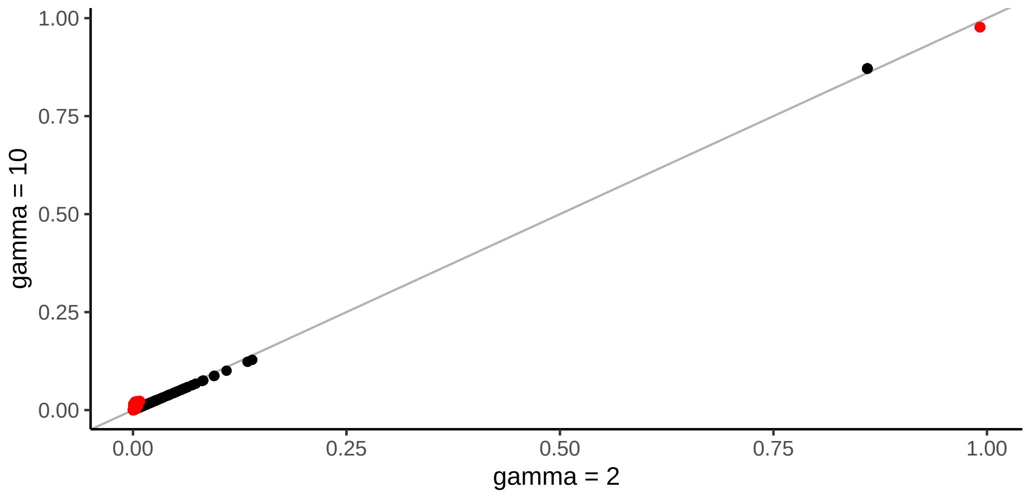

Supplementary Figure 36: Estimated flows using gravity model. The x-axis shows the flows using a uniform prior for γ with upper limit 2 and the y-axis shows the flows using a uniform prior varying from 1 to 10. The black dots show the flows estimated using the first 21 days of incidence data from ProMED. Flows estimated using parameters fitted to the first 210 days of incidence data are shown in red. Results are shown for the model with calibration window set to 14 days.

8.1 Forecasts using ProMED data

8.1.1 Calibration window of 2 weeks

8.1.2 Forecast horizon 4 weeks

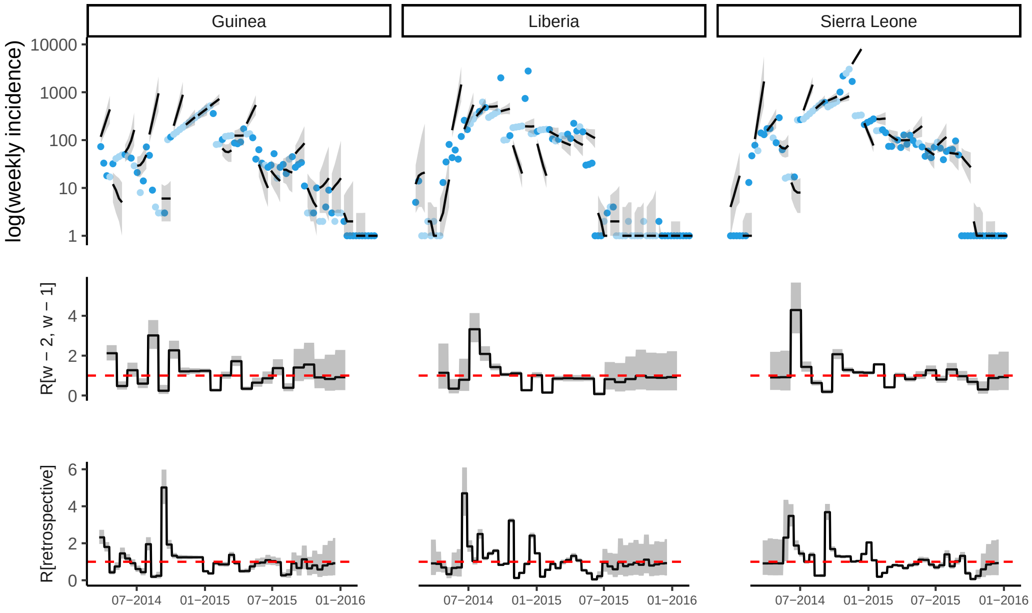

Supplementary Figure 37: Observed and predicted incidence, and reproduction number estimates from ProMED data. The calibration window is 2 weeks and the forecast horizon is 4 weeks.

8.1.3 Forecast horizon 6 weeks

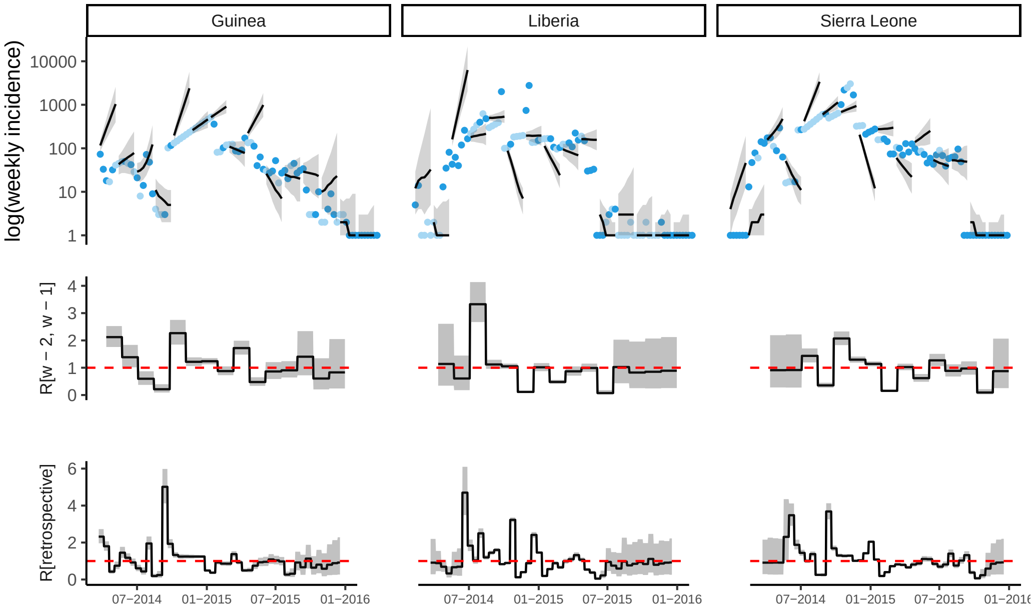

Supplementary Figure 38: Observed and predicted incidence, and reproduction number estimates from ProMED data.The calibration window is 2 weeks and the forecast horizon is 6 weeks.

8.1.4 Forecast horizon 8 weeks

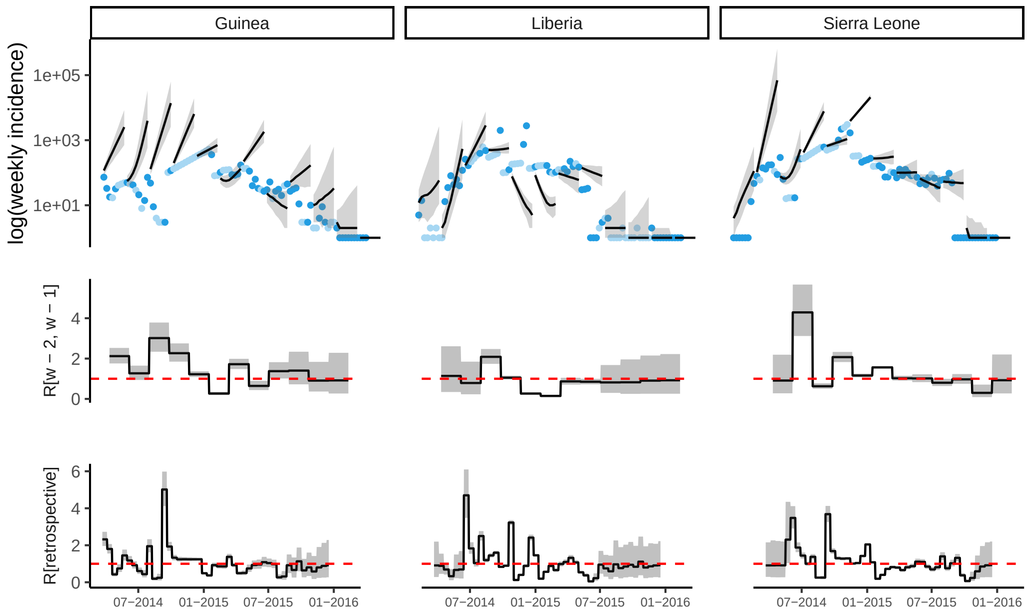

Supplementary Figure 39: Observed and predicted incidence, and reproduction number estimates from ProMED data.The calibration window is 2 weeks and the forecast horizon is 8 weeks.

8.1.5 Calibration window of 4 weeks

8.1.6 Forecast horizon 4 weeks

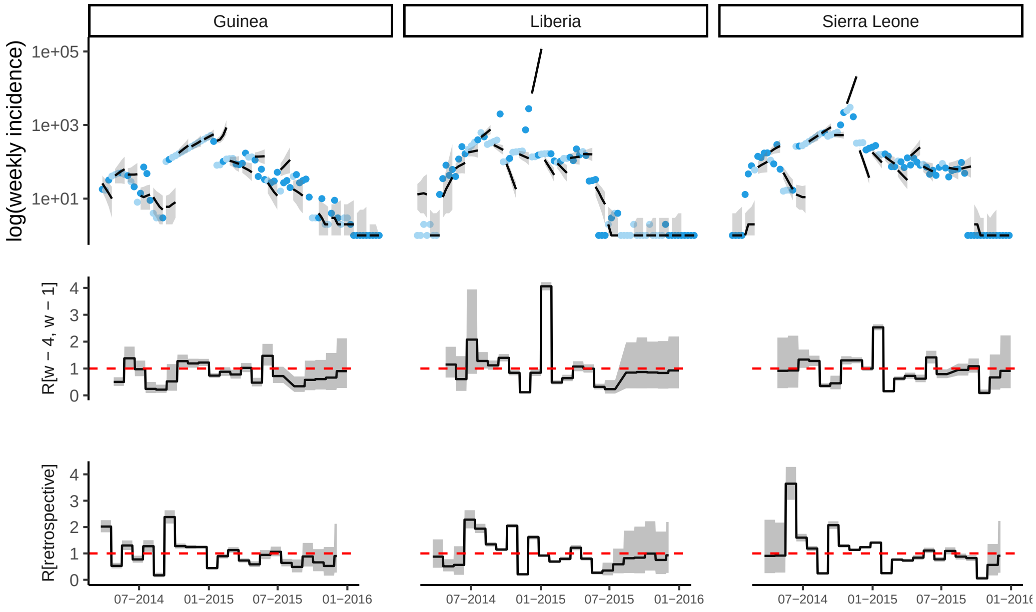

Supplementary Figure 40: Observed and predicted incidence, and reproduction number estimates from ProMED data.The calibration window is 4 weeks and the forecast horizon is 4 weeks.

8.1.7 Forecast horizon 6 weeks

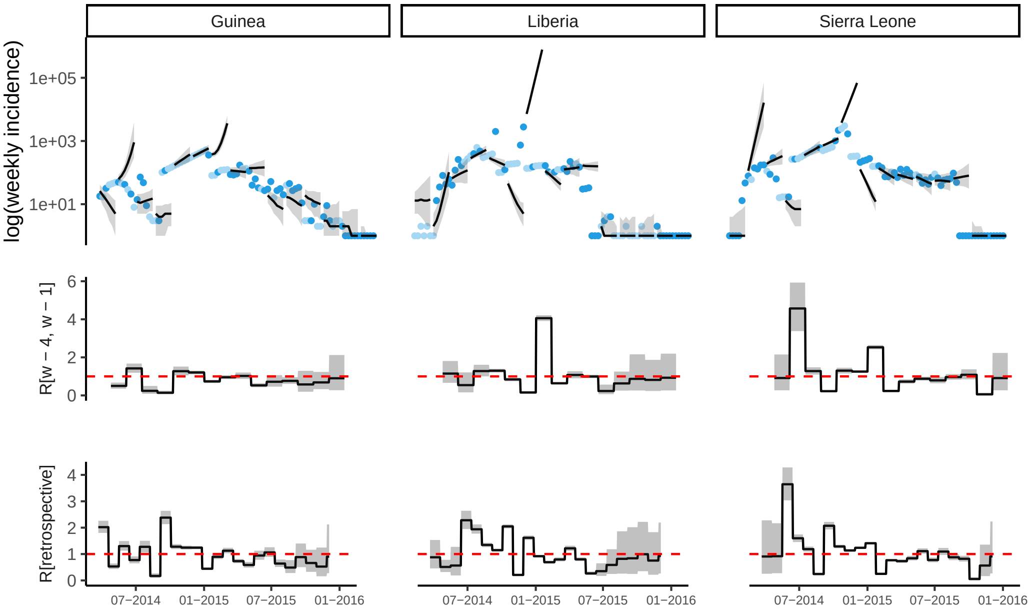

Supplementary Figure 41: Observed and predicted incidence, and reproduction number estimates from ProMED data.The calibration window is 4 weeks and the forecast horizon is 6 weeks.

8.1.8 Forecast horizon 8 weeks

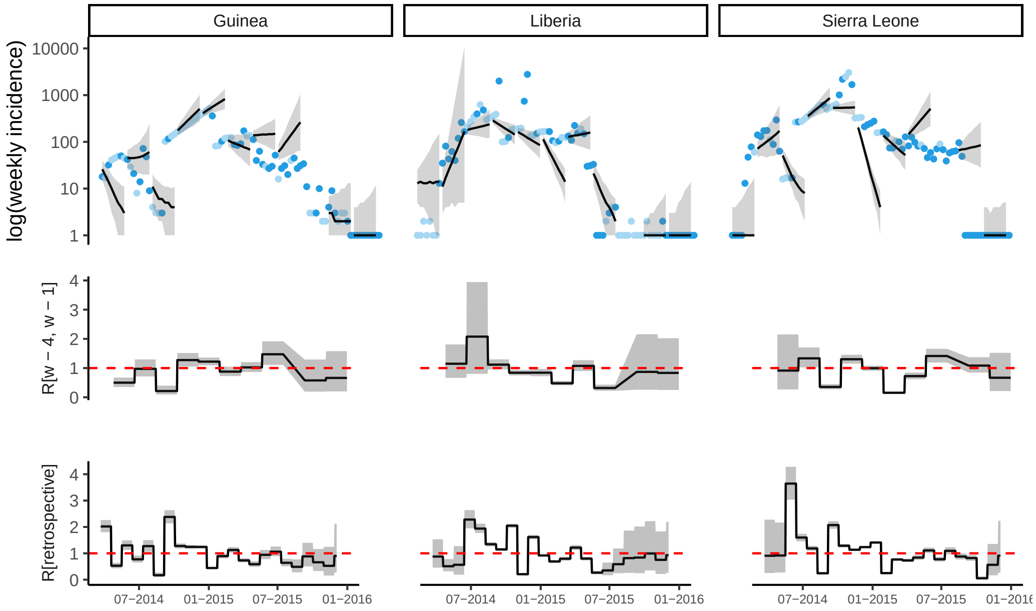

Supplementary Figure 42: Observed and predicted incidence, and reproduction number estimates from ProMED data.The calibration window is 4 weeks and the forecast horizon is 8 weeks.

8.1.9 Calibration window of 6 weeks

8.1.10 Forecast horizon 4 weeks

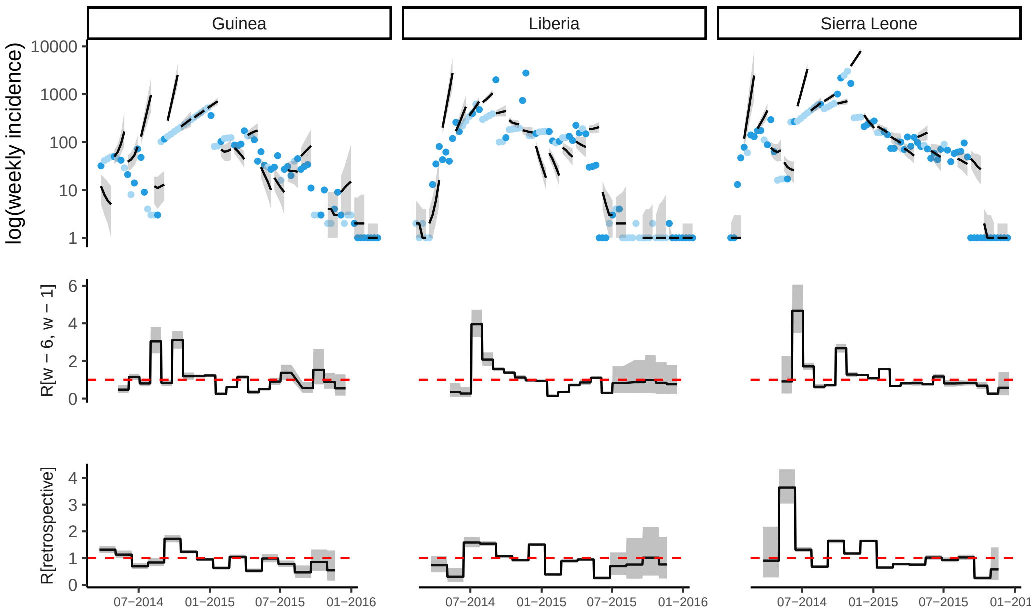

Supplementary Figure 43: Observed and predicted incidence, and reproduction number estimates from ProMED data.The calibration window is 6 weeks and the forecast horizon is 4 weeks.

8.1.11 Forecast horizon 6 weeks

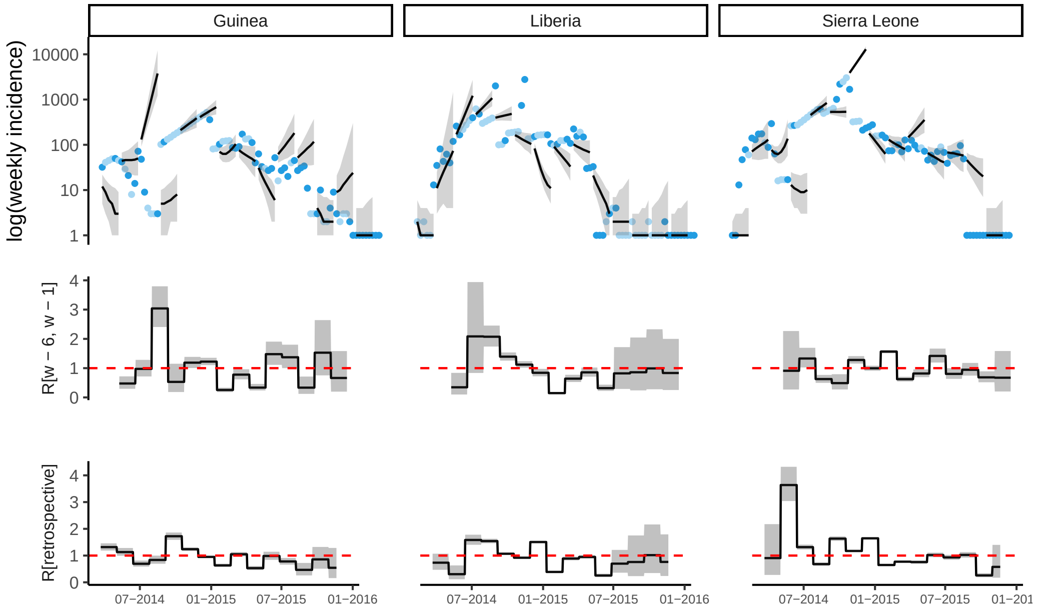

Supplementary Figure 44: Observed and predicted incidence, and reproduction number estimates from ProMED data.The calibration window is 6 weeks and the forecast horizon is 6 weeks.

8.1.12 Forecast horizon 8 weeks

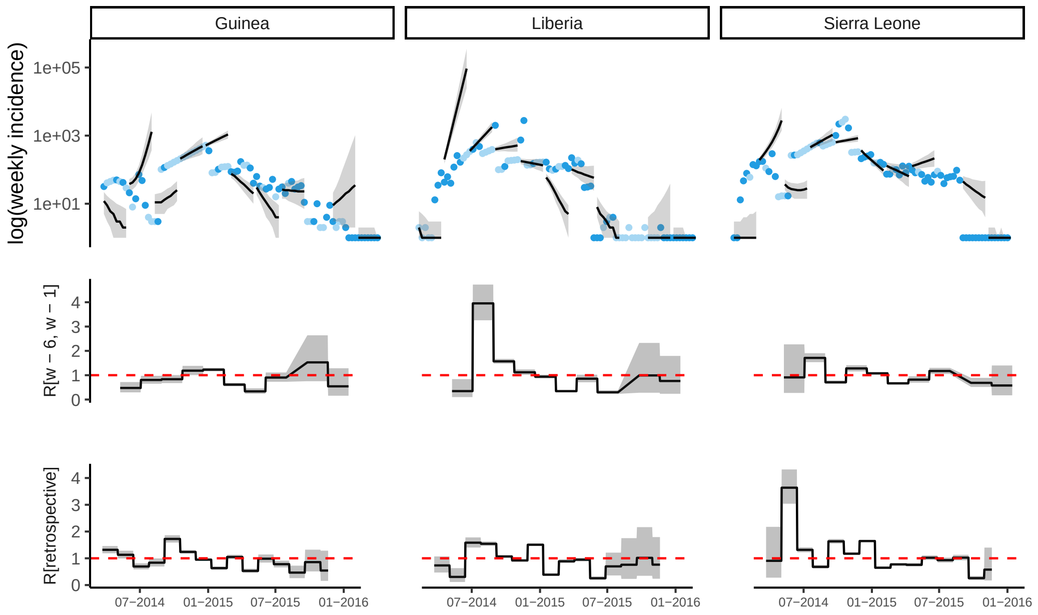

Supplementary Figure 45: Observed and predicted incidence, and reproduction number estimates from ProMED data.The calibration window is 6 weeks and the forecast horizon is 8 weeks.

8.2 Model performance with alternate priors for mobility model parameter

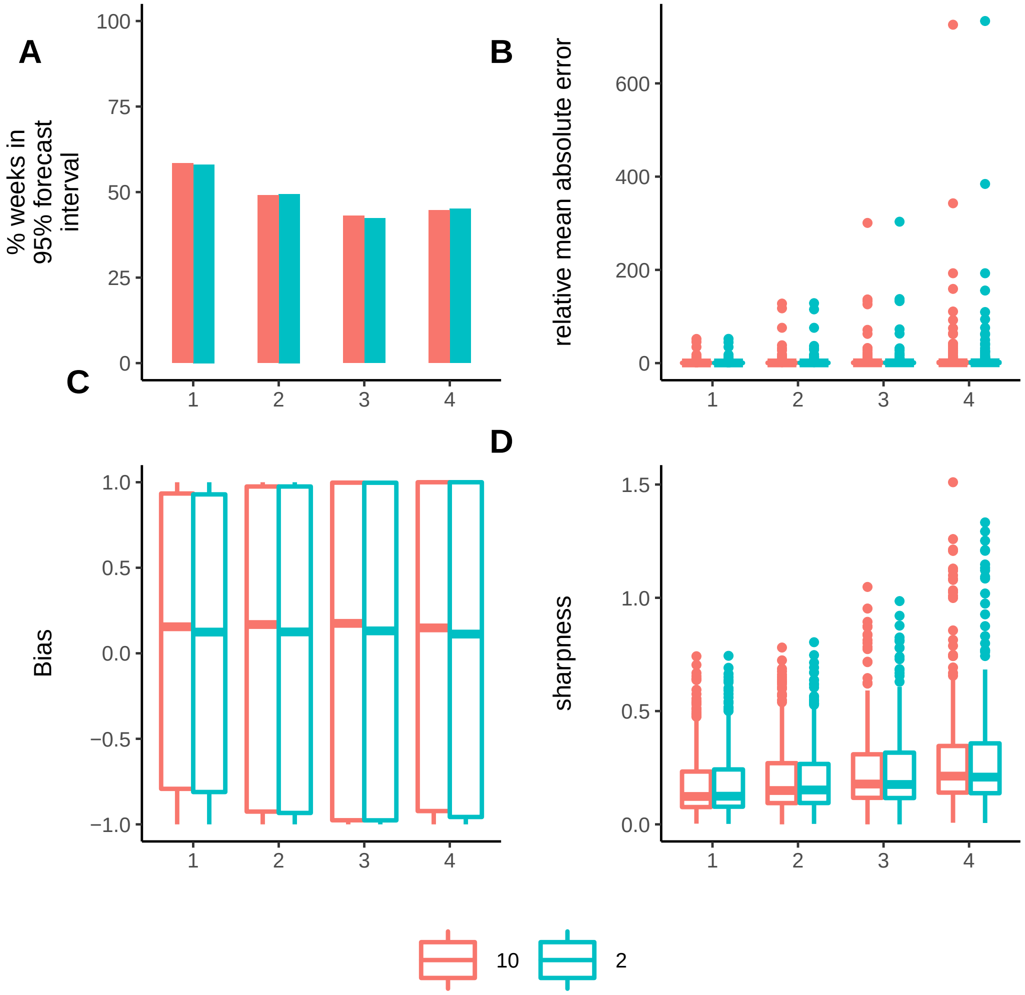

Supplementary Figure 46: Model performance metrics allowing γ to vary up to 2 or 10. The performance metrics (A) the percentage of weeks for which the 95% forecast interval contained the observed incidence, (B) relative mean absolute error, (C) bias and (D) sharpness.

9 Risk of spatial spread

In this section, we present additional analyses carried out on predicting the spatial spread of the epidemic. Classification of alerts raised 1, 2, 3, and 4 weeks ahead are shown in Supplementary Figure 47. We also assessed the sensitivity of the model when the analysis was restricted to countries other than Guinea, Liberia and Sierra Leone (Supplementary Figure 48). At 93% threshold, the model exhibited high specificity (83.3%) and sensitivity (82.0%) in predicting presence of cases in weeks following a week with no observed cases in each country (Supplementary Figure 49). In predicting presence of cases in countries with no or low incidence, or in a week following a week in which no cases were observed, the sensitivity improved at higher thresholds with a reasonably low false alert rate (Supplementary Figure 50). Finally, we find that the model is able to attain a high sensitivity and specificity relatively early in the epidemic using all three data sources (Supplementary Figure 51). Since WHO data used here were only available for Guinea, Liberia and Sierra Leone, we affixed data from ProMED for all countries other than these three to WHO data for the purpose of classifying alerts.

For a given threshold (x^{th} percentile of the forecast interval), we defined a True alert for a week where the xth percentile of the forecast interval and the observed incidence for a country were both greater than 0; false alert for a week where the threshold for a country was greater than 0 but the observed incidence for that country was 0; and missed alert for a week where the threshold for a country was 0 but the observed incidence for that country was greater than 0. True alert rate is the ratio of correctly classified true alerts to the total number of true and missed alerts (i.e., (true alerts)/(true alerts + missed alerts)). False alert rate is similarly the ratio of false alerts to the total number of false alerts and weeks of no alert (where the observed and the threshold incidence are both 0).

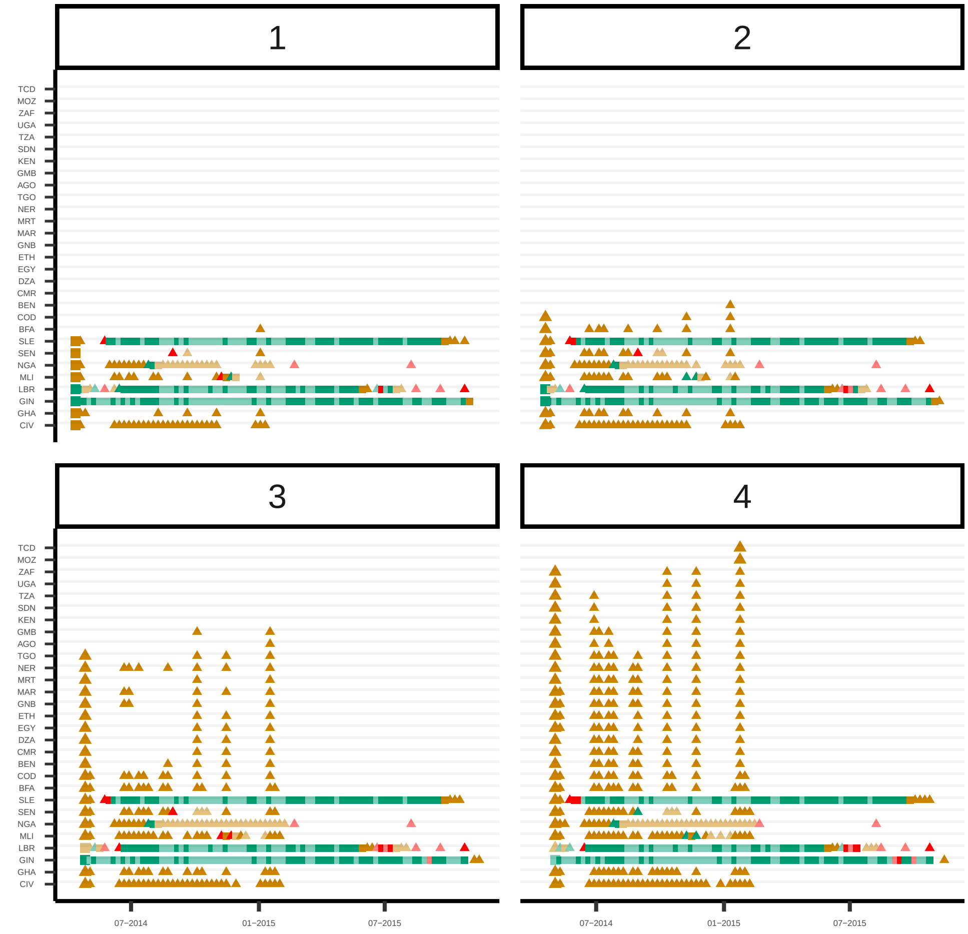

Supplementary Figure 47: Predicted weekly presence of cases in each country up to 4 weeks ahead. The panels shows the True (green), False (orange) and Missed (red) 1, 2, 3 and 4 week ahead alerts using the 42.5^{th} percentile of the forecast interval as threshold. The figure only shows countries on the African continent for which either the 42.5^{th} percentile of the forecast interval or the observed incidence was greater than 0 at least once. The first alert in each country is shown using larger symbols (square or triangle). Alerts in a country in a week where there were no observed cases in the previous week are shown using hollow triangles. In each case, weeks for which all observed points were imputed are shown in lighter shades. Country codes, shown on the y-axis, are as follows: AGO - Angola, BEN - Benin, BFA - Burkina Faso, CIV - Côte d'Ivoire, CMR - Cameroon, COD - Democratic Republic of Congo, DZA - Algeria, EGY - Egypt, ETH - Ethiopia, GHA - Ghana, GIN - Guinea, GMB - Gambia, GNB - Guinea-Bissau, KEN - Kenya, LBR - Liberia, MAR - Morocco, MLI - Mali, MOZ - Mozambique, MRT - Mauritania, NER - Niger, NGA - Nigeria, SDN - Sudan, SEN - Senegal, SLE - Sierra Leone, SSD - South Sudan, TCD - Chad, TGO - Togo, TUN - Tunisia, TZA - Tanzania, UGA - Uganda, ZAF - South Africa. The alerts are based on forecasts using the ProMED data, a 2-week calibration window and a 4 week forecast horizon.

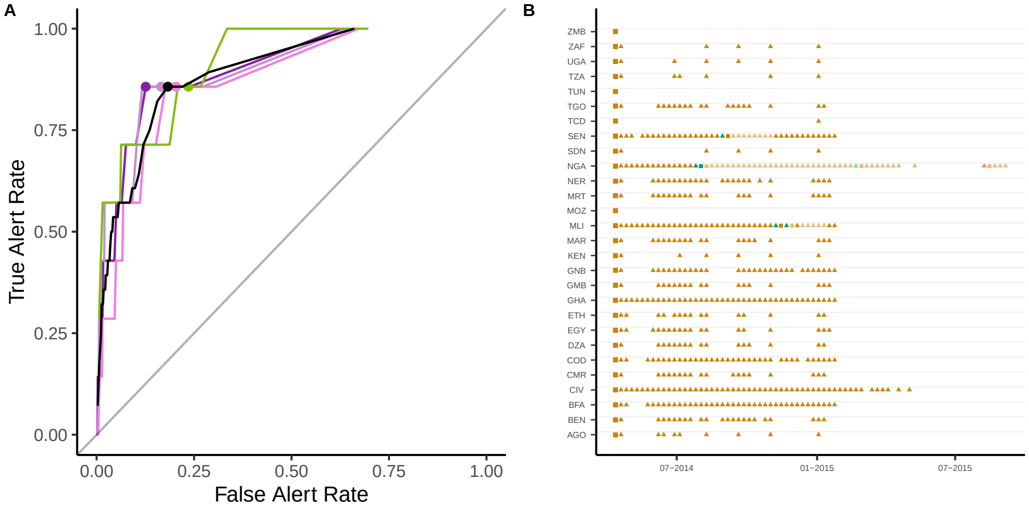

Supplementary Figure 48: Predicted weekly presence of cases in countries other than the three majorly affected countries (Guinea, Liberia and Sierra Leone). The left panel shows the True and False alert rates using different thresholds for classification for alerts raised 1 (violet), 2 (light violet), 3 (dark pink) and 4 (light green) weeks ahead. The black curve depicts the overall True and False alert rates. On each curve, the dot shows the True and False Alert rates at 92.5% threshold. The right panel shows the True (green), False (orange) and Missed (red) 1 week ahead alerts using the 92.5th percentile of the forecast interval as threshold. The figure only shows countries on the African continent for which either the 92.5th percentile of the predicted incidence or the observed incidence was greater than 0 at least once. The first alert in each country is shown using larger symbols (square or triangle). Alerts in a country in a week where there were no observed cases in the previous week are shown using hollow triangles. In each case, weeks for which all observed points were imputed are shown in lighter shades. Country codes, shown on the y-axis, are as follows: AGO - Angola, BEN - Benin, BFA - Burkina Faso, CIV - Côte d'Ivoire, CMR - Cameroon, COD - Congo - Kinshasa, DZA - Algeria, EGY - Egypt, ETH - Ethiopia, GHA - Ghana, GMB - Gambia, GNB - Guinea-Bissau, KEN - Kenya, MAR - Morocco, MLI - Mali, MRT - Mauritania, NER - Niger, NGA - Nigeria, SDN - Sudan, SEN - Senegal, TGO - Togo, TZA - Tanzania, UGA - Uganda, ZAF - South Africa, MOZ - Mozambique, MWI - Malawi, SSD - South Sudan, TCD - Chad, TUN - Tunisia, BDI - Burundi, CAF - Central African Republic, COG - Congo - Brazzaville, LBY - Libya, MDG - Madagascar, RWA - Rwanda, ZMB - Zambia, ZWE - Zimbabwe, ERI - Eritrea, GAB - Gabon, SOM - Somalia, CPV - Cape Verde, GNQ - Equatorial Guinea, NAM - Namibia. The alerts are based on forecasts using the ProMED data, a 2-week calibration window and a 4 week forecast horizon.

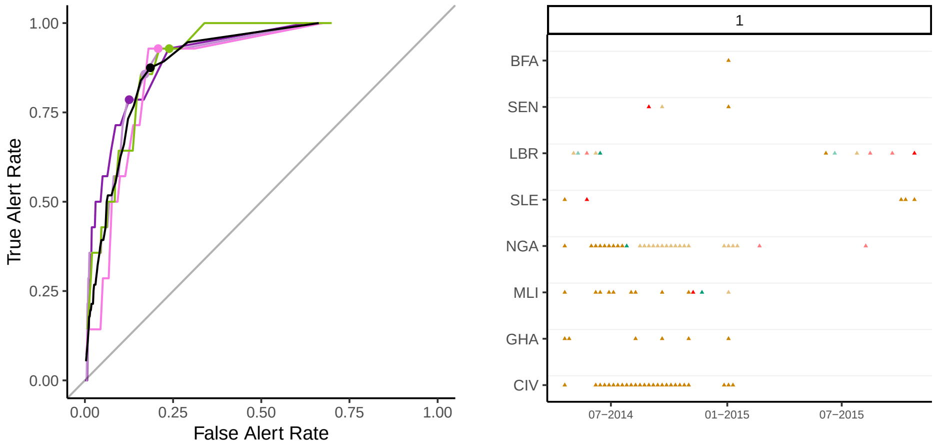

Supplementary Figure 49: Predicted weekly presence of cases for each country in weeks following a week with no observed cases. The left panel shows the True and False alert rates using different thresholds for classification for alerts raised 1 (violet), 2 (light violet), 3 (dark pink) and 4 (light pink) weeks ahead. The black curve depicts the overall True and False alert rates. On each curve, the dot shows the True and False Alert rates at 93% threshold. For a given threshold (x^{th} percentile of the forecast interval), we defined a True alert for a week where the xth percentile of the forecast interval and the observed incidence for a country were both greater than 0; false alert for a week where the threshold for a country was greater than 0 but the observed incidence for that country was 0; and missed alert for a week where the threshold for a country was 0 but the observed incidence for that country was greater than 0. True alert rate is the ratio of correctly classified true alerts to the total number of true and missed alerts (i.e., (true alerts)/(true alerts + missed alerts)). False alert rate is similarly the ratio of false alerts to the total number of false alerts and weeks of no alert (where the observed and the threshold incidence are both 0). The right panel shows the True (green), False (orange) and Missed (red) 1 week ahead alerts using the 93rd percentile of the forecast interval as threshold. The figure only shows countries on the African continent for which either the 93rd percentile of the predicted incidence or the observed incidence was greater than 0 at least once. The first alert in each country is shown using larger symbols (square or triangle). Alerts in a country in a week where there were no observed cases in the previous week are shown using hollow triangles. In each case, weeks for which all observed points were imputed are shown in lighter shades. Country codes, shown on the y-axis, are as follows: BFA - Burkina Faso, CIV - Côte d'Ivoire, GHA - Ghana, GIN - Guinea, LBR - Liberia, MLI - Mali, NGA - Nigeria, SEN - Senegal, SLE - Sierra Leone. The alerts are based on forecasts using the ProMED data, a 2-week calibration window and a 4 week forecast horizon.

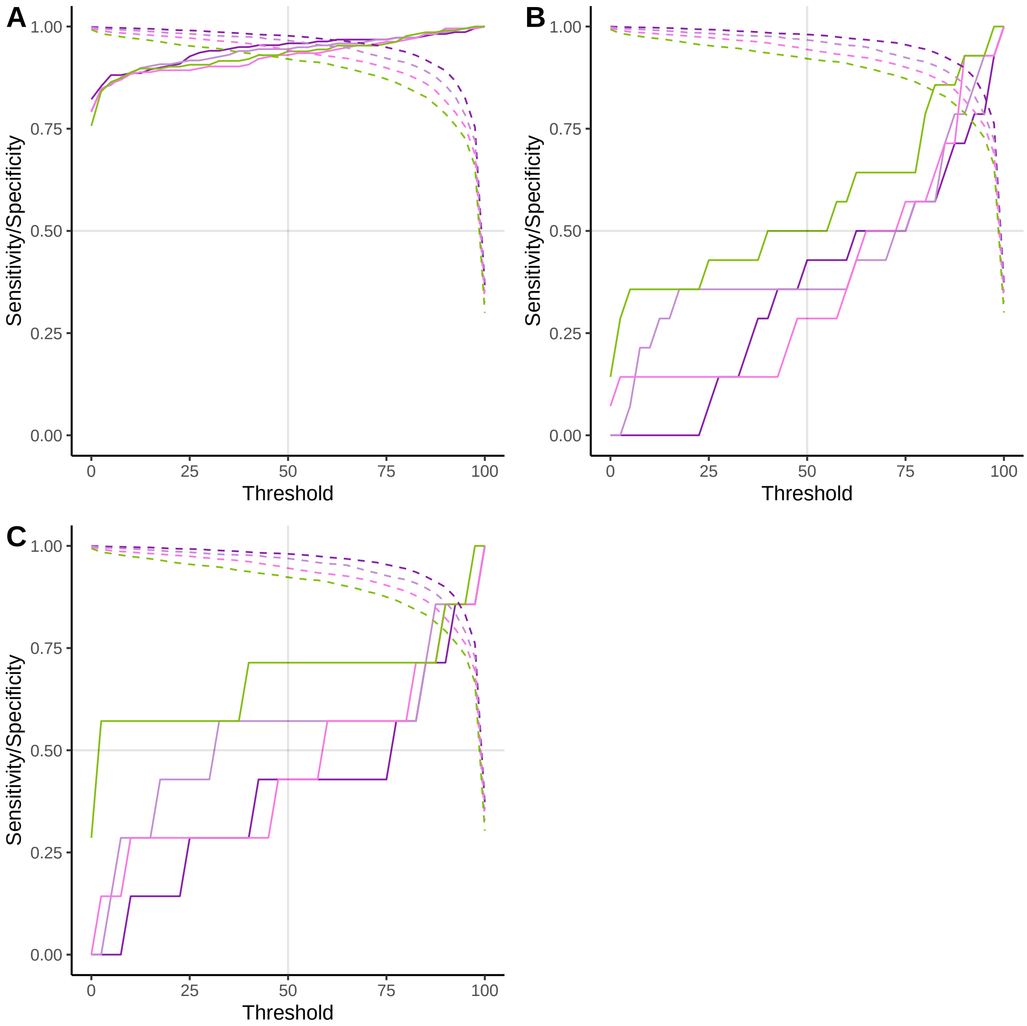

Supplementary Figure 50: Sensitivity and specificity at various thresholds for (A) all countries in Africa (B) all countries in Africa except Guinea, Liberia and Sierra Leone, and (C) all in Africa in weeks following a week with no observed cases. The solid and dashed lines depict the sensitivity and specificity respectively of 1 (violet), 2 (light violet), 3 (pink) and 4 (light green) week ahead alerts.

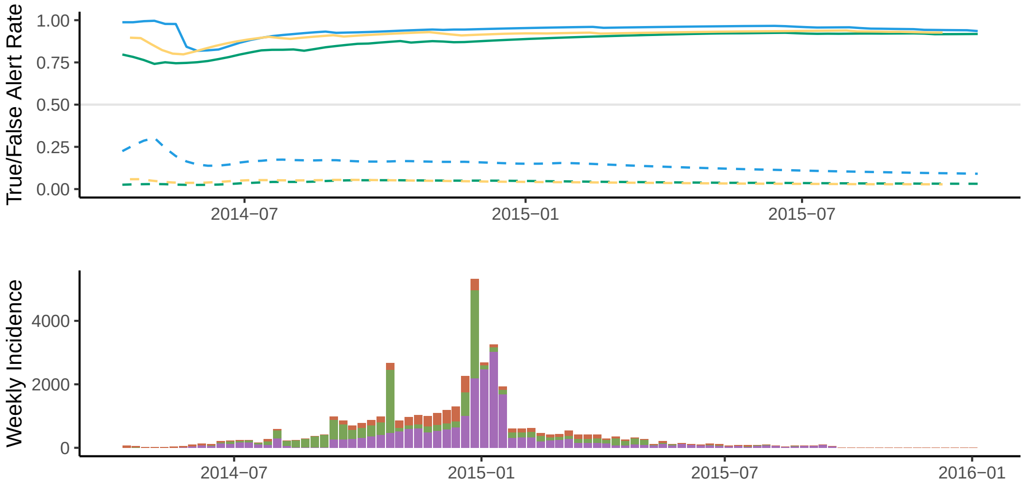

Supplementary Figure 51: True and False alert rates at 50% threshold using ProMED (blue), HealthMap (green) and WHO (yellow) data over the course of the epidemic. The solid lines show the True Alert rate and the dashed lines show the False alert rate averaged over the 4 weeks forecast horizon. The bottom panel shows the weekly incidence for Guinea (deep orange), Liberia (green) and Sierra Leone (violet) obtained from ProMED data.

10 Model Convergence Diagnostic Report

$$
\begin{array}{l}
\begin{array}{r}
\\
\text{Window 1}\left\{\begin{array}{c}\\ \\ \\ \end{array}\right. \\
\text{Window 2}\left\{\begin{array}{c}\\ \\ \\ \end{array}\right.
\end{array}
\begin{array}{c}
\begin{array}{ccccc}
\overbrace{\quad}^{1} & \overbrace{\quad}^{2} & \overbrace{\quad}^{3} & \overbrace{\qquad\qquad}^{4} & \overbrace{\qquad\qquad}^{5}
\end{array} \\
\begin{bmatrix}
R[0] & R[1] & R[2] & R[3] & \dots & R[6] & R[7] & \dots & R[54] \\
R[0] & R[1] & R[2] & R[3] & \dots & R[6] & R[7] & \dots & R[54] \\
\dots & & & & & & & & \\
R[0] & R[1] & R[2] & R[3] & \dots & R[6] & R[7] & \dots & R[54] \\
R[55] & R[56] & R[57] & R[58] & \dots & R[61] & R[62] & \dots & R[109] \\
R[55] & R[56] & R[57] & R[58] & \dots & R[61] & R[62] & \dots & R[109] \\
\dots & & & & & & & & \\
R[55] & R[56] & R[57] & R[58] & \dots & R[61] & R[62] & \dots & R[109]
\end{bmatrix}
\end{array}
\end{array}
$$

Figure 1: An example matrix of time-varying reproduction numbers in the model for 55 countries and 2 time windows. This matrix illustrates the underlying implementation. Reproduction number was only estimated for countries with non-zero incidence at any point during the epidemic.

For estimating the time-varying reproduction number for each country, we split the duration of the total outbreak into intervals of equal width. We assume that transmissibility in each location stays constant within each time window and thus, within a time window, we estimated a single reproduction number for each country with non-zero incidence, dividing the 55 countries on African continent into 5 groups. Each country in a group was forced to have the same reproduction number in each time window (Fig 1). The first three groups correspond of the three mainly affected countries - Sierra Leone, Guinea and Liberia. The countries that shared a border with these three countries were grouped together. These were Mali, Cte d'Ivoire, Guinea-Bissau and Gambia. The rest of the countries were assigned to the fifth group. The other parameters in the model were p_{stay} and γ (See Methods). We used the R package ggmcmc to assess the convergence of the fitted model. The rest of this document presents an example report produced by ggmcmc. For more details, reader is referred to the package documentation at `http://xavier-fim.net/post/using_ggmcmc/`.

Supplementary References

[1] John G Saw, Mark CK Yang, and Tse Chin Mo. Chebyshev Inequality with Estimated Mean and Variance. *The American Statistician*, 38(2):130–132, 1984.
